# scLM: Automatic Detection of Consensus Gene Clusters Across Multiple Single-cell Datasets

**DOI:** 10.1016/j.gpb.2020.09.002

**Published:** 2020-12-24

**Authors:** Qianqian Song, Jing Su, Lance D. Miller, Wei Zhang

**Affiliations:** 1 Center for Cancer Genomics and Precision Oncology, Wake Forest Baptist Comprehensive Cancer Center, Wake Forest Baptist Medical Center, Winston Salem, NC 27157, USA; 2 Department of Cancer Biology, Wake Forest School of Medicine, Winston Salem, NC 27157, USA; 3 Department of Biostatistics, Indiana University School of Medicine, Indianapolis, IN 46202, USA

**Keywords:** Single-cell RNA sequencing, Consensus clustering, Latent space, Markov Chain Monte Carlo, Maximum likelihood approach

## Abstract

In gene expression profiling studies, including **single-cell RNAsequencing** (scRNA-seq) analyses, the identification and characterization of co-expressed genes provides critical information on cell identity and function. Gene co-expression clustering in scRNA-seq data presents certain challenges. We show that commonly used methods for single-cell data are not capable of identifying co-expressed genes accurately, and produce results that substantially limit biological expectations of co-expressed genes. Herein, we present single-cell Latent-variable Model (scLM), a gene co-clustering algorithm tailored to single-cell data that performs well at detecting gene clusters with significant biologic context. Importantly, scLM can simultaneously cluster multiple single-cell datasets, *i.e.*, **consensus clustering**, enabling users to leverage single-cell data from multiple sources for novel comparative analysis. scLM takes raw count data as input and preserves biological variation without being influenced by batch effects from multiple datasets. Results from both simulation data and experimental data demonstrate that scLM outperforms the existing methods with considerably improved accuracy. To illustrate the biological insights of scLM, we apply it to our in-house and public experimental scRNA-seq datasets. scLM identifies novel functional gene modules and refines cell states, which facilitates mechanism discovery and understanding of complex biosystems such as cancers. A user-friendly R package with all the key features of the scLM method is available at https://github.com/QSong-github/scLM.

## Introduction

Co-expressed genes work in concert in biological pathways and processes [1], [2], [3]. Such genes are involved in crucial biological activities including immune cell activation [4], [5], cellular epithelial-mesenchymal transition (EMT) [6], and transcription factor-mediated gene regulatory networks and signaling pathways [7], [8]. Co-expression of genes based on similarities among their expression profiles, has been a primary way to unravel gene-gene relationships and facilitate functional annotation [9], [10], [11], [12]. Therefore, identification of co-expressed genes provides functional insights into underlying cellular and molecular mechanisms in normal and disease processes.

The recently developed single-cell RNA sequencing (scRNA-seq) technology provides high resolution of gene expression at the single-cell level [13], yet presents certain challenges for gene expression analysis [14]. In contrast to bulk RNA-seq, single-cell data have been shown to exhibit a characteristic negative binomial (NB) distribution pattern [15], [16], [17], [18], wherein genes suffer from stochastic dropouts and over-dispersion problems. Dropouts, or genes that exhibit excessive zero values [19], [20], [21], represent a special type of missing value, which can be caused by low RNA input or stochastic expression fluctuation at the single-cell level. Over-dispersion relates to the substantially large cell-to-cell variability in gene expression profiles which likely arises from technical noise stemming from low input RNA and PCR amplification bias [22].

Rapid advances in scRNA-seq technologies have made it feasible to perform population-scale studies in which the transcriptome is measured for thousands of single cells from multiple samples or conditions [23], [24], [25], [26], [27]. This in turn has amplified the need for versatile gene co-expression approaches that not only address the unique challenges of scRNA-seq data, but also the challenges of dataset integration including batch effects, technical variations (*e.g.*, mRNA quality and pre-amplification efficiency), and extrinsic biological variabilities.

Classical methods designed for analysis of bulk transcriptome data such as weighted gene co-expression network analysis (WGCNA) [28] and Clust [29] are not designed to account for the unique characteristics of scRNA-seq data. Some network-based approaches for single-cell data, including Single-Cell rEgulatory Network Inference and Clustering (SCENIC) [30], Cell Specific Network (CSN) [31], and Left Truncated Mixture Gaussian (LTMG) [32], can detect gene co-expression modules as part of the network reconstruction. However, these methods do not account for the technical noise and extrinsic variance among multiple samples. Therefore, there is a clear need to develop a tailored and effective method for scRNA-seq data to extract “consensus” co-expressed genes [11], that is, to extract the genes that are consistently co-expressed in each of the multiple datasets.

Herein, we have developed a novel method, single-cell Latent-variable Model (scLM), to simultaneously extract co-expressed genes that exhibit consensus behaviors from multiple single-cell datasets. The scLM method accounts for both cell-level covariates and sample-level batch effects. We assessed the performance of scLM in both simulated data and experimental data. scLM achieves the best performance over other commonly used methods. We then applied scLM to our in-house scRNA-seq data generated from four non-small cell lung cancer (NSCLC) tumor tissues and their corresponding adjacent normal tissues. The scLM method identified tumor-specific co-expressed gene modules with significant prognostic values. Furthermore, these co-expression modules contributed to the subtle characterization of lung tumor cell states. In addition, we applied scLM to analyze a set of malignant cells from NSCLC, head and neck squamous cell carcinoma (HNSCC), and melanoma. We discovered a common co-expressed gene program across different cancer types, providing insights into fundamental mechanisms of carcinogenesis.

## Method

### scLM

We proposed a latent-variable model to explicitly disentangle different sources of variabilities in population-scale scRNA-seq data. Our goal was to perform simultaneous detection of co-expressed genes across multiple single-cell conditions/datasets. Specifically, let $x_{\mathrm{ijk}}$ denotes the gene expression level experimentally measured for the i-th gene $\left(i\in \left\{1,\cdots ,m\right\}\right)$ in the j-th cell $\left(j\in \left\{1,\cdots ,n_{k}\right\}\right)$ in condition/dataset $k\left(k\in \left\{1,\cdots ,K\right\}\right)$.

As multiple recent studies [15], [16], [17], [18] showed that the expression of most genes in single-cell data is sufficiently captured by NB distribution, NB model is chosen as an appropriate model to formulate single-cell data. It is supported by the physical modeling of bursting gene expression [18], [33] and is also commonly used in scRNA-seq analysis [15], [16], [17], [18]. Therefore, without loss of generality, we assumed that the measured gene expression $x_{\mathrm{ijk}}$ for cell j in dataset k follows the NB distribution NB(p,γ), which has the probability function as:

(1) $$f\left(X=x_{\mathrm{ijk}},p,\gamma \right)=\frac{\Gamma \left(x_{\mathrm{ijk}}+\gamma \right)}{\Gamma \left(\gamma \right)\Gamma \left(1+x_{\mathrm{ijk}}\right)}{\left(1-p\right)}^{\gamma }p^{x_{\mathrm{ijk}}}$$

If μ, θ, and $\sigma^{2}$ represent the mean, dispersion, and variance of this NB distribution, then we have

(2) $$\mu =\frac{p\gamma }{1-p},\;\theta =\gamma ,\;\sigma^{2}=\frac{p\gamma }{({1-p)}^{2}}$$

also,

(3) $$p=\frac{\mu }{\mu +\theta },\;\gamma =\theta$$

Therefore, the probability function converts to:

(4) $$f\left(x_{\mathrm{ijk}};\theta ,u\right)=\frac{\Gamma \left(x_{\mathrm{ijk}}+\theta \right)}{\Gamma \left(\theta \right)\Gamma \left(1+x_{\mathrm{ijk}}\right)}{\left(\frac{\theta }{\theta +u}\right)}^{\theta }{\left(\frac{u}{\theta +u}\right)}^{x_{\mathrm{ijk}}}$$

As u and θ are regarding different genes $\left(i\in \left\{1,\cdots ,m\right\}\right)$ and batches $\left(k\in \left\{1,\cdots ,K\right\}\right)$, we have

(5) $$f\left(x_{\mathrm{ijk}};\theta_{\mathrm{ik}},u_{\mathrm{ik}}\right)=\frac{\Gamma \left(x_{\mathrm{ijk}}+\theta_{\mathrm{ik}}\right)}{\Gamma \left(\theta_{\mathrm{ik}}\right)\Gamma \left(1+x_{\mathrm{ijk}}\right)}{\left(\frac{\theta_{\mathrm{ik}}}{\theta_{\mathrm{ik}}+u_{\mathrm{ik}}}\right)}^{\theta_{\mathrm{ik}}}{\left(\frac{u_{\mathrm{ik}}}{\theta_{\mathrm{ik}}+u_{\mathrm{ik}}}\right)}^{x_{\mathrm{ijk}}}$$

Herein, $u_{\mathrm{ik}}$ represents the estimation for the intrinsic gene expression level across all cells in sample k, $\theta_{\mathrm{ik}}$ is the dispersion parameter, and $\sigma_{\mathrm{ik}}^{2}=u_{\mathrm{ik}}+\frac{{u_{\mathrm{ik}}}^{2}}{\theta_{\mathrm{ik}}}$ represents the square deviation of the observed gene expression level across cells in this sample.

Let $z_{i}={\left(z_{i1},\cdots ,z_{i\lambda }\right)}^{\text{'}}$ be a vector consisting of λ unobserved latent variables that are shared by K different datasets. We assumed the generalized linear model (GLM) below

(6) $$u_{\mathrm{ik}}\sim \alpha_{\mathrm{jk}}+\beta_{\mathrm{jk}}z_{i}$$

which was used to distinguish the intrinsic biological signals $z_{i}$ from the extrinsic variabilities ($\alpha_{\mathrm{jk}}$ and $\beta_{\mathrm{jk}}$) including the technical variances at the cell level (j) and batch effects at the sample level (k). That is, the $u_{\mathrm{ik}}$ is composed of the intrinsic biological signals of gene i captured by latent variables $z_{i}$ regardless of the confounding variabilities at the cell level and sample level, while variances due to technical biases and batch effects are captured by offsets $\alpha_{\mathrm{jk}}$ and scale factors $\beta_{\mathrm{jk}}$. Since $z_{i}$ is the same for specific gene i, and $u_{\mathrm{ik}}$ is estimated from observed counts $x_{\mathrm{ijk}}$, we further turned the formula into

(7) $$u\left(x_{\mathrm{ijk}}\text{|}z_{i}\right)\sim \alpha_{\mathrm{jk}}+\beta_{jk}z_{i}$$

**Figure 1:**
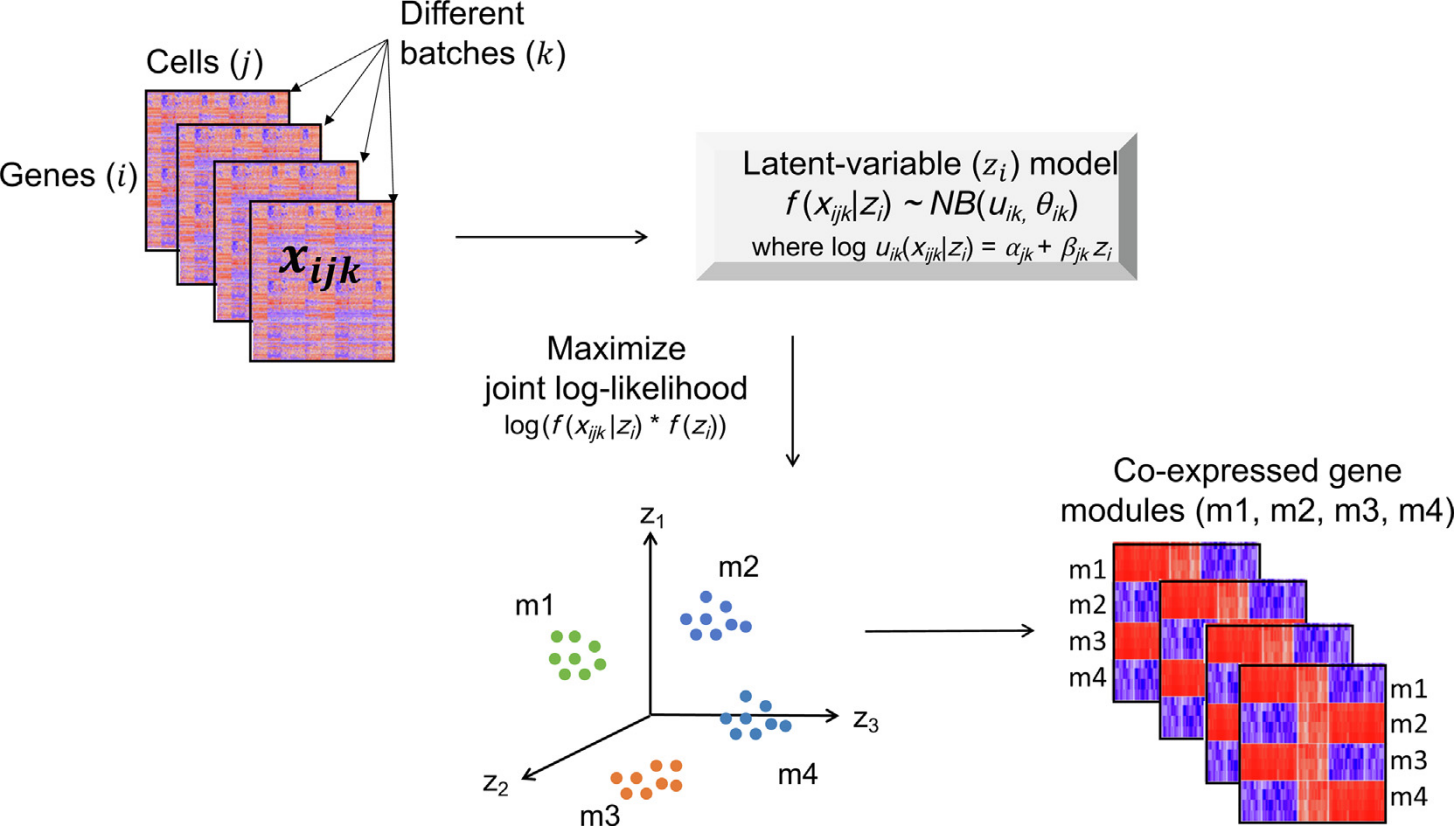
**Schematicof scLM for identifying consensus co-expressed gene clusters across multiple datasets** Schematic representation of how consensus co-expressed genes across multiple datasets could be discovered by scLM. The gene expression profiles of individual cells were disentangled by the latent variables representing the intrinsic biological signals, and the related coefficients reflecting the technical variances. scLM, single-cell Latent-variable Model.

To alleviate the impact of extreme values, we utilized logarithm form in the linear model that has been frequently used [34], [35], [36], [37], [38] in scRNA-seq data, *i.e.*, the GLM,

(8) $$\log u\left(x_{\mathrm{ijk}}|z_{i}\right)=\alpha_{\mathrm{jk}}+\beta_{\mathrm{jk}}z_{i}$$

where $u\left(x_{\mathrm{ijk}}|z_{i}\right)$ is the conditional mean of $x_{\mathrm{ijk}}$ given $z_{i}$. In this way, the original gene expression data were projected into a λ-dimensional latent space Z by the GLM, with the technical biases and batch effects removed during the projection. In this latent space, the expression level of gene i is represented as $z_{i}$. Since genes sharing similar expression patterns are located close to each other, a group of co-expressed genes will form a cluster in the latent space. Thus, different groups of co-expressed gene modules can be identified through clustering of the latent variables (Figure 1).

To estimate the parameters in our model, we used the maximum likelihood approach. As is assumed above that $x_{\mathrm{ijk}}$ follows the N*B* distribution, the conditional log-likelihood function of $x_{\mathrm{ijk}}$ can be written as:

(9) $$\log\;f\;\left(x_{\mathrm{ijk}}|z_{i},\alpha_{\mathrm{jk}},\beta_{\mathrm{jk}},\theta_{\mathrm{ik}}\right)=\frac{\Gamma \left(\theta_{\mathrm{ik}}+x_{\mathrm{ijk}}\right)}{\Gamma \left(\theta_{\mathrm{ik}}\right)\Gamma \left(1+x_{\mathrm{ijk}}\right)}{\left(\frac{u_{\mathrm{ik}}}{\theta_{\mathrm{ik}}+u_{\mathrm{ik}}}\right)}^{x_{\mathrm{ijk}}}{\left(\frac{\theta_{\mathrm{ik}}}{\theta_{\mathrm{ik}}+u_{\mathrm{ik}}}\right)}^{\theta_{\mathrm{ik}}}$$

in which,

$$u_{\mathrm{ik}}=\exp\left(\alpha_{\mathrm{jk}}+\beta_{\mathrm{jk}}z_{i}\right)$$

For the latent variable $z_{i}$, $f(z_{i})$ represents the density function of the standard multivariate normal distribution N(0, $I_{\lambda }$). Therefore, the joint log-likelihood of ($x_{\mathrm{ijk}}$, $z_{i}$) can be written as

(10) $$l\left(x_{\mathrm{ijk}},z_{i};\alpha_{\mathrm{jk}},\beta_{\mathrm{jk}}\right)=\sum_{i=1}^{m}\sum_{j=1}^{n_{k}}\sum_{k=1}^{K}\left\{\log\;f\;\left(x_{\mathrm{ijk}}|z_{i},\alpha_{\mathrm{jk}},\beta_{\mathrm{jk}}\right)+\log\;f\;\left(z_{i}\right)\right\}$$

To control model complexity and overfitting, we applied the least absolute shrinkage and selection operator (LASSO, L1-norm penalty), to the following penalized joint log-likelihood estimation:

$$l\left(x_{\mathrm{ijk}},z_{i};\alpha_{\mathrm{jk}},\beta_{\mathrm{jk}}\right)-\overset{K}{\underset{k=1}{\sum }}\overset{n_{k}}{\underset{j=1}{\sum }}\partial_{k}\left|\right|\beta_{\mathrm{jk}}{\left|\right|}_{1}$$

Then the above parameters are estimated by maximizing the penalized joint log-likelihood function, that is, maximizing the following penalized joint log-likelihood,

(11) $$\underset{\alpha_{\mathrm{jk}},\beta_{\mathrm{jk}}}{\max}l\left(x_{\mathrm{ijk}},z_{i},\alpha_{\mathrm{jk}},\beta_{\mathrm{jk}}\right)-\sum_{k=1}^{K}\sum_{j=1}^{n_{k}}\partial_{k}\left|\right|\beta_{\mathrm{jk}}{\left|\right|}_{1}=\underset{\alpha_{\mathrm{jk}},\beta_{\mathrm{jk}}}{max}\overset{m}{\underset{i=1}{\sum }}\overset{n_{k}}{\underset{j=1}{\sum }}\overset{K}{\underset{k=1}{\sum }}\left\{\log\;f\;\left(x_{\mathrm{ijk}}|z_{i},\alpha_{\mathrm{jk}},\beta_{\mathrm{jk}}\right)+\log\;f\;\left(z_{i}\right)\right\}-\overset{K}{\underset{k=1}{\sum }}\overset{n_{k}}{\underset{j=1}{\sum }}\partial_{k}\left|\right|\beta_{\mathrm{jk}}{\left|\right|}_{1}$$

where the summation is due to the conditional independence assumption of $x_{\mathrm{ijk}}$ given $z_{i}$.

To estimate the parameters $\alpha_{\mathrm{jk}}$ and $\beta_{\mathrm{jk}}$, we solved the following optimization problem conditional on $z_{i}$,

(12) $$\underset{\alpha_{\mathrm{jk}},\beta_{\mathrm{jk}}}{min}\overset{m}{\underset{i=1}{\sum }}\overset{n_{k}}{\underset{j=1}{\sum }}\overset{K}{\underset{k=1}{\sum }}\log\;f\;\left(x_{\mathrm{ijk}}|z_{i},\alpha_{\mathrm{jk}},\beta_{\mathrm{jk}}\right)+\overset{K}{\underset{k=1}{\sum }}\overset{n_{k}}{\underset{j=1}{\sum }}\partial_{k}\left|\right|\beta_{\mathrm{jk}}{\left|\right|}_{1}$$

here we used the coordinate descent algorithm provided in [39], therefore optimized the above log-likelihood function. Herein the update of parameters $\alpha_{\mathrm{jk}}$ and $\beta_{\mathrm{jk}}$ depend on $z_{i}$. As the latent variables $z_{i}$ were not observable in our model, we used the Markov Chain Monte Carlo (MCMC) simulation to iteratively update $z_{i}$, for maximizing the penalized joint log-likelihood. That is, we replaced the value in the parameter updates by its expectation with respect to $z_{i}$, through repeatedly sampling the latent variables $z_{i}$ from the following joint posterior distribution, *i.e.*,

$$f\left(z_{i}\right)\overset{n_{k}}{\underset{j=1}{\prod }}\overset{K}{\underset{k=1}{\prod }}f\left(x_{\mathrm{ijk}}|z_{i},\alpha_{\mathrm{jk}},\beta_{\mathrm{jk}}\right)$$

With the estimated latent variables $z_{i}$, that is, with the genes projected into the latent space, we clustered genes that were projected in the latent space to identify co-expressed genes. Here we used K-means clustering to divide genes into λ clusters based on the latent variables $z_{i}$. The parameter λ can be either determined according to the Bayesian information criterion (BIC), or chosen by user’s preference.

### Data generation in simulation studies

Based on the single-cell data characteristics, we used the NB distribution to simulate two synthetic cohorts (synthetic cohort 1 and synthetic cohort 2). Each synthetic cohort contained 9 sets of simulated gene expression data with an increasing number of datasets (D1–D9). That is, D1 contained one individual dataset (*n* = 1), D2 contained two individual datasets (*n* = 2), …, and D9 contained nine individual datasets (*n* = 9). Each individual dataset contained 180 genes belonging to three clusters, with 60 co-expressed genes in each of the three clusters. For each gene cluster c∈{1,2,3} in batch n, their gene expression was sampled from the NB distribution $\mathrm{NB}(u_{\mathrm{cn}},\theta_{\mathrm{cn}})$, where $u_{\mathrm{cn}}$ and $\theta_{\mathrm{cn}}$ referred to the mean and deviation, respectively. Different gene clusters had different values of $u_{\mathrm{cn}}$ and $\theta_{\mathrm{cn}}$. Full expression values and cluster membership for these datasets were provided in the scLM example data.

Additionally, we utilized the Splatter package [40] to generate another two synthetic cohorts (synthetic cohort 3 and synthetic cohort 4) of simulated data with dropout effects, which more accurately recapitulated actual scRNA-seq data distributions. Specifically, we adjusted the batch parameters “batch.facLoc” and “batch.facScale” as 1 and generated 16 different batches of data. Each batch consisted of 240 cells, and 240 genes constituting four groups of co-expressed genes as the group truth, which was achieved by adjusting the “de.prob” parameter. We also added the dropout effects in these simulation data by setting “experiment” for global dropout and the “dropout.mid” parameter. These 16 batches of data made up the synthetic cohorts 3 and 4. Full expression values were provided in the scLM example data.

### In-house and public single-cell data

#### In-house dataset

Fresh tumor and adjacent normal tissues from four NSCLC patients were collected by the Tumor Tissue and Pathology Shared Resource (TTPSR) into Miltenyi Tissue Storage Medium (Catalog No. 130-100-008, Miltenyi Biotec, San Diego, CA). Tissues were then processed to single-cell suspensions using the Miltenyi Human Tumor Dissociation Kit (Catalog No. 130-095-929, Miltenyi Biotec) and the gentleMACS Octo Dissociator with Heaters (Catalog No. 130-096-427, Miltenyi Biotec). Red blood cells were removed by negative selection using Miltenyi CD235a (Glycophorin A) microbeads (Catalog No. 130-050-501, Miltenyi Biotec) and LS Columns (Catalog No. 130-042-401, Miltenyi Biotec). Recovered cell numbers were determined by trypan blue exclusion using a LUNA II automated cell counter (Catalog No. L40001, Logos Biosystems, Annandale, VA). In preparation for scRNA-seq, cells were thawed and washed according to the demonstrated protocol developed for human peripheral blood mononuclear cells (PBMCs) by 10X Genomics (San Francisco, CA).

All scRNA-seq procedures were performed by the Cancer Genomics Shared Resource (CGSR) of the Wake Forest Baptist Medical Center Comprehensive Cancer Center (WFBMC-CCC). Viable cells in suspensions were loaded into wells of a 10X Genomics Chromium Single Cell A Chip Kit (Catalog No. PN-120236, 10X Genomics). Single-cell gel beads in emulsion (GEMs) were created on a Chromium Single Cell Controller and scRNA-seq libraries were prepared using the Chromium Single Cell 3′ Library and Gel Bead Kit v2 (Catalog No. PN-120237, 10X Genomics). Sequencing libraries were loaded at 1.3 PM on an Illumina NextSeq500 with a High Output 150 cycle Kit (Catalog No. FC-404-2002, Illumina, San Diego, CA) for paired-end sequencing. A total of 11,813 single cells were captured, with the number of cells recovered per channel ranging from 369 to 2502. Low-quality cells were discarded if the cell with expressed genes was smaller than 200. Only malignant cells from four tumor samples and epithelial cells from three adjacent normal samples were used in this study. The scRNA-seq data were deposited in the Gene Expression Omnibus (GEO) of National Center for Biotechnology Information (NCBI) database (GEO: GSE117570) at https://onlinelibrary.wiley.com/doi/full/10.1002/cam4.2113 [41].

#### Melanoma dataset

We downloaded the expression matrix data of melanoma from the GEO of NCBI database (GEO: GSE72056) at https://www.ncbi.nlm.nih.gov/pubmed/27124452 [42]. This dataset included expression profiles of 23,689 genes in 4645 cells from 19 melanoma tumors. These cells included both malignant cancer cells and non-malignant cells. For the input matrix of scLM, a sample is excluded if it contains < 200 cells, and a gene is excluded from the input matrix if it is expressed in < 300 cells.

#### HNSCC dataset

We downloaded the expression matrix data of the HNSCC dataset from the GEO of NCBI database (GEO: GSE103322) at https://www.sciencedirect.com/science/article/pii/S0092867417312709 [6]. This dataset consisted of 5902 cells from 18 patient samples after initial quality controls, including 2215 malignant and 3363 non-malignant cells. For our analyses, we used the samples with more than 200 malignant cells and genes expressed in over 300 cells as the input matrix.

#### Breast cancer dataset

We downloaded the expression matrix data of breast cancer (BR) scRNA-seq dataset from the GEO of NCBI database (GEO: GSE118390) at https://www.nature.com/articles/s41467-018–06052-0 [23]. For our analysis, we used malignant cells and genes expressed in over 300 cells as input.

### Clustering evaluation index

Each clustering result produced by different methods was assessed using clustering evaluation indices, including the Adjusted Rand Index (ARI) [43], the Calinski-Harabasz (CH) index [44], the Davies-Bouldin (DB) index [45], and the Dunn index [46]. CH index evaluated the cluster validity based on the average between- and within-cluster sum of squares. DB index was obtained by averaging all the cluster similarities. Dunn index used the minimum pairwise distance between objects in different clusters as the inter-cluster separation and the maximum diameter among all clusters as the intra-cluster compactness. Larger CH index, smaller DB index, and larger Dunn index represented better clustering results.

### Cell clustering based on co-expressed gene modules

With the co-expressed gene modules, we utilized mean value of the modules in each single cell as input for graph-based clustering. Uniform manifold approximation and projection (UMAP) was used to visualize cell clusters. Graph-based clustering was performed using the Seurat package (v3.1), and UMAP analysis was performed using the “umap” package (v.0.2.3.1) [47] in R (v.3.4.3). The number of epochs (n_epochs) was set at 20. The n_neighbors value was set at 15, and min_dist was set as 0.1.

### Statistical analysis

Kaplan-Meier (KM) analysis was performed using the “survival” R package (http://cran.r-project.org/web/packages/survival/index.html). Log-rank test was used to test the differences of survival curves. When evaluating the performance of scLM, *P* value was calculated by *t*-test.

### Functional analysis

#### Hallmark collection

We downloaded the Hallmark gene set collection for functional analyses from Molecular Signatures Database (MSigDB) [48], which was a widely used and comprehensive database. Each hallmark in this collection consisted of a “refined” gene set that conveyed a specific biological state or process and displayed coherent expression. The hallmarks effectively summarized most of the relevant information of the original founder sets and, by reducing both variation and redundancy, provided more refined and concise inputs for gene set enrichment analysis.

#### Pathway database

Reactome (http://www.reactome.org) was a manually curated open-data resource of human pathways and reactions, which was an archive of biological processes and a tool for discovering potential functions. Gene sets derived from the Reactome [49] and Kyoto Encyclopedia of Genes and Genomes (KEGG) [50] pathway database were downloaded from the MSigDB Collections.

#### Enrichment test

Functional enrichment based on the Reactome and Gene Ontology (GO) databases was assessed by hypergeometric test, which was used to identify a priori-defined gene set that showed statistically significant differences between two given clusters. Enrichment test was performed by the clusterProfiler package [51]. Test *P* values were further adjusted by Benjamini-Hochberg correction, and adjusted *P* values less than 0.05 were considered statistically significant.

## Results and discussion

### Overview of scLM

We developed a new method, scLM, for simultaneously identifying consensus co-expressed genes from multiple scRNA-seq datasets. Our hypothesis was that co-expressed genes coordinating biological processes could be captured across multiple different datasets. In our model, we assumed that latent variables captured the intrinsic signals of the co-expressed genes regardless of technical variances and batch effects among different datasets. Figure 1 provided an illustrative overview of the scLM method. Briefly, the input contained a collection of multiple datasets (k) representing the single-cell sequencing data generated under different clinical or experimental settings. In the k-th dataset, we assumed that the observed expression levels, $x_{\mathrm{ijk}}$, of the i-th gene across cells $j\in \{1,\cdots ,n_{k}\}$ followed the NB distribution $\mathrm{NB}(u_{\mathrm{ik}},\theta_{\mathrm{ik}})$. The intrinsic biological variability of gene i across all cells and all datasets was captured by the latent variables $z_{i}$ in a λ-dimension latent space. This was achieved through a conditional GLM $\log\;u\left(x_{\mathrm{ijk}}|z_{i}\right)=\alpha_{\mathrm{jk}}+\beta_{\mathrm{jk}}z_{i}$ that distinguished the intrinsic biological variability $z_{i}$ from the extrinsic signals ($\alpha_{\mathrm{jk}}$ and $\beta_{\mathrm{jk}}$) including the technical variances at the cell level (j) and batch effects at the sample level (k). The latent variables and other parameters were estimated and obtained using MCMC and maximum likelihood approaches. Therefore, different groups of co-expressed genes (m1–m4) across multiple datasets were identified through clustering genes in the latent space. Further explanations of the mathematical model were included in the Method.

### Performance evaluation on simulation data

To evaluate the performance of scLM, we benchmarked it against other methods, including LTMG [32], CSN [31], Seurat-wgcna, MNN-wgcna, and SCENIC [30]. Seurat-wgcna and MNN-wgcna referred to the co-expression analysis using WGCNA [28], following the batch correction by Seurat [52] or MNN [53]. As SCENIC relied on the RcisTarget database that required real gene input, we omitted comparing with SCENIC on simulation data but still included it in the comparison on real single-cell data.

We first generated two synthetic data cohorts (synthetic cohorts 1 and 2) from NB distribution. Each cohort contained 9 sets (D1–D9) of simulated data with an increasing number of samples. That is, D1 contained one individual dataset (*n* = 1), D2 contained two individuals of datasets (*n* = 2), and so on. Each set contained three co-expressed gene clusters as ground truth. Additionally, we utilized the Splatter package [40] to generate another two batches of simulated data (synthetic cohorts 3 and 4) with dropout effects, which could more accurately recapitulate actual scRNA-seq data distributions. Details of the simulation datasets were provided in the Method.

**Figure 2:**
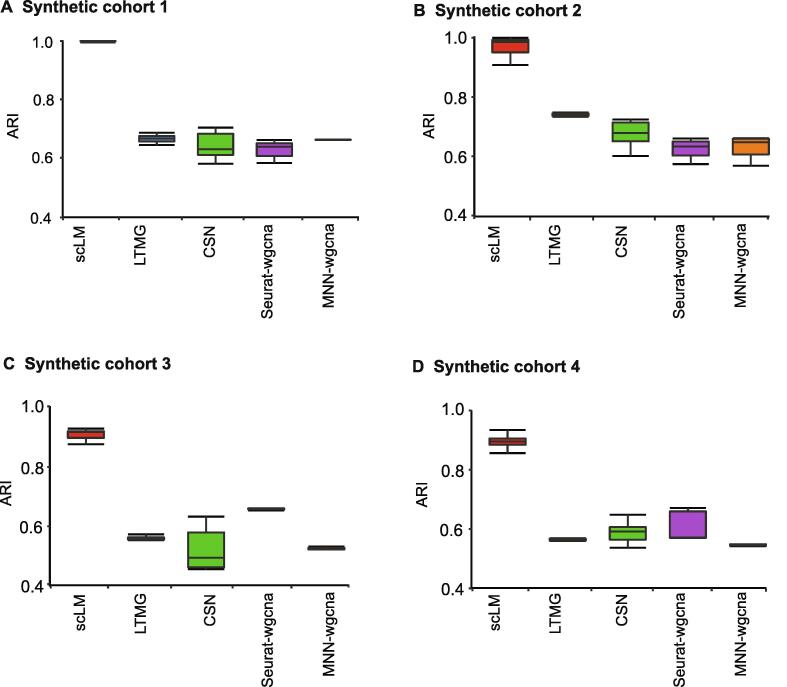
**Performance evaluation on simulation data** scLM was compared with other methods (LTMG, CSN, Seurat-wgcna, and MNN-wgcna) on four synthetic cohorts. Each synthetic cohort contains  9 sets of simulated data with an increasing number of samples. The bar plot represents the ARI of identified gene clusters compared to the ground truth. **A.** ARI of synthetic cohort 1. **B.** ARI of synthetic cohort 2. **C.** ARI of synthetic cohort 3. **D.** ARI of synthetic cohort 4. ARI, Adjusted Rand Index; LTMG, Left Truncated Mixture Gaussian; CSN, Cell Specific Network.

With the simulated data cohorts, we applied scLM and other methods (LTMG, CSN, Seurat-wgcna, and MNN-wgcna) to identify the co-expression clusters. To assess and quantify clustering accuracy, we used the ARI [43] as the performance metric to rank these methods (Figure 2). The corresponding bar plots represented the ARI of the identified clusters by each method compared to the ground truth. Notably, scLM accurately identified each gene cluster in four cohorts, and demonstrated much higher ARIs (mean ± SE: 0.979 ± 0.063 for synthetic cohort 1; 0.971 ± 0.031 for synthetic cohort 2; 0.899 ± 0.043 for synthetic cohort 3; 0.886 ± 0.025 for synthetic cohort 4). The other methods showed relatively lower ARIs. For example, CSN showed lower ARIs in synthetic cohort 1 (mean ± SE: 0.627 ± 0.028) and synthetic cohort 3 (mean ± SE: 0.520 ± 0.070). LTMG presented little higher ARIs and lower variances in four synthetic cohorts. These results demonstrated the outperformance of scLM in identifying accurate co-expressed genes from multiple datasets.

### Evaluation of scLM using experimental data

To further demonstrate the performance of scLM, we compared scLM with other methods (LTMG, CSN, Seurat-wgnca, MNN-wgcna, and SCENIC) on experimental scRNA-seq datasets. For comparisons, we used two in-house datasets from lung tumor and adjacent normal tissues as well as three public datasets from BR, HNSCC, and melanoma. The data pre-processing procedures were described in the Method.

**Figure 3:**
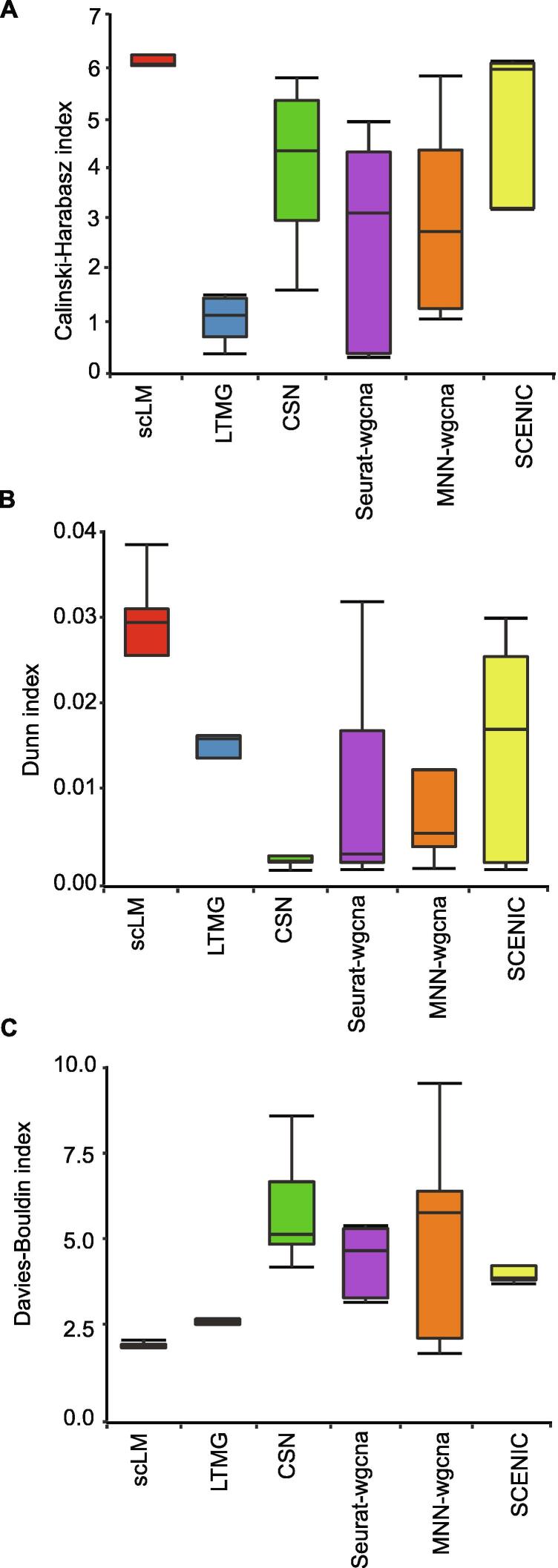
**Evaluation of scLM using experimental data** scLM was compared with other methods (LTMG, CSN, Seurat-wgcna, MNN-wgcna, and SCENIC) on five experimental datasets. Multiple evaluation indices were used, including: the Calinski-Harabasz index (**A**), the Dunn index (**B**), and the Davies-Bouldin index (**C**).

To assess and quantify clustering accuracy on real datasets, we used performance metrics including the CH index [44], Dunn index [46], and DB index [45], to rank these methods. Importantly, scLM produced sets of clusters that showed significantly higher CH values than other methods (Figure 3A), especially higher than LTMG (*P* = 1.75E−07) and MNN-wgcna (*P* = 0.02), demonstrating that scLM achieved better cluster validity than other methods based on average between- and within-cluster sum of squares. In addition, compared to other methods, scLM also achieved significantly higher Dunn index scores representing better inter-cluster separation and intra-cluster compactness (Figure 3B), and lower DB index scores reflecting higher cluster quality (Figure 3C). Though SCENIC and Seurat-wgcna showed higher Dunn index score in one dataset (HNSCC), they failed to show superior performance on other datasets. Thus scLM proved to achieve the best partitioning of co-expressed gene clusters that are most distinct from each other.

### scLM identified co-expressed genes with significantly enriched biological functions

**Figure 4:**
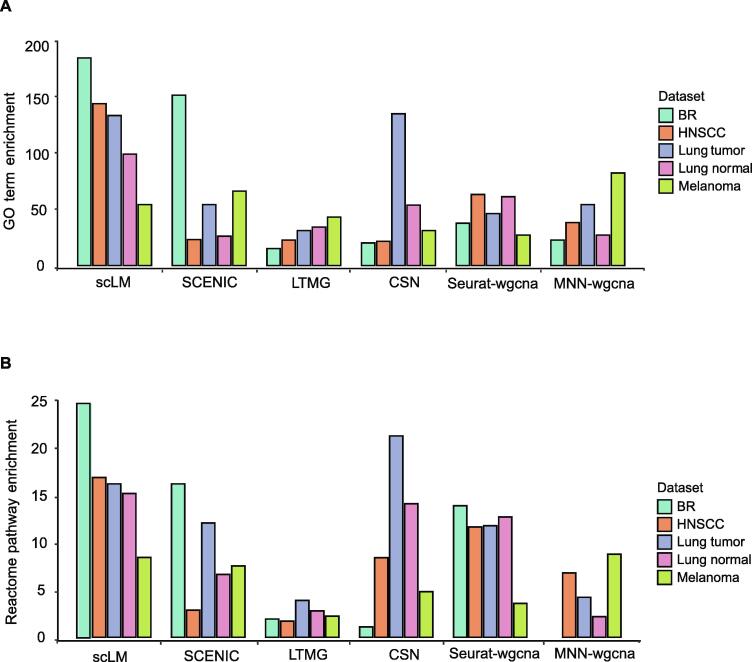
**scLM identifies co-expressed genes with significantly enriched biological functions** **A.** The average number of significantly enriched GO terms (adjusted *P* value < 0.05) based on the co-expressed genes identified by different methods. **B.** The average number of significantly enriched pathways in Reactome database (adjusted *P* value < 0.05) based on the co-expressed genes identified by different methods. GO, Gene Ontology.

As co-expressed genes were likely to be enriched with biological functions, we compared the extent to which different methods affected the functional discovery, based on their identified co-expressed genes. First, the aforementioned methods were evaluated for their capability to detect enriched GO terms in the five experimental datasets. Different methods identified gene clusters enriched with different GO enrichment results. The average number of significantly enriched GO terms (adjusted *P* value < 0.05) ranged from 15 to 184 (Figure 4A). scLM extracted co-expressed genes with more enriched functional terms than other methods in three of the five datasets (*i.e.*, the BR, HNSCC, and lung Normal datasets). SCENIC identified relatively high number of enriched GO terms in the BR dataset, whereas low number of enriched GO terms in other datasets; CSN identified relatively high number of enriched GO terms in the lung Tumor dataset but low number of enriched GO terms in other datasets; MNN-wgcna identified relatively high number of enriched GO terms in the melanoma dataset but low number of enriched GO terms in other datasets. LTMG and Seurat-wgcna showed lower number of enriched GO terms in five datasets. Similar results were observed when we strengthened the enriched significance by the adjusted *P* value < 0.01 (Figure S1). The number of significant terms became fewer for all the methods, yet scLM identified the most on all datasets except for the melanoma dataset. Some methods, like LTMG, failed to identify gene clusters with enriched terms at the threshold of adjusted *P* value < 0.01.

In addition to GO terms, we also examined the enriched pathways in the Reactome database, based on the co-expressed genes identified by different methods (Figure 4B). Different methods showed different pathway enrichment results. Importantly, scLM identified co-expressed genes with more enriched pathways than other methods in three of the five datasets (*i.e.*, the BR, HNSCC, and lung Normal datasets). Taken together, these results demonstrated that scLM outperforms other methods in functional discovery of co-expressed genes.

### scLM identified the tumor-specific modules enriched in specific cell state

**Figure 5:**
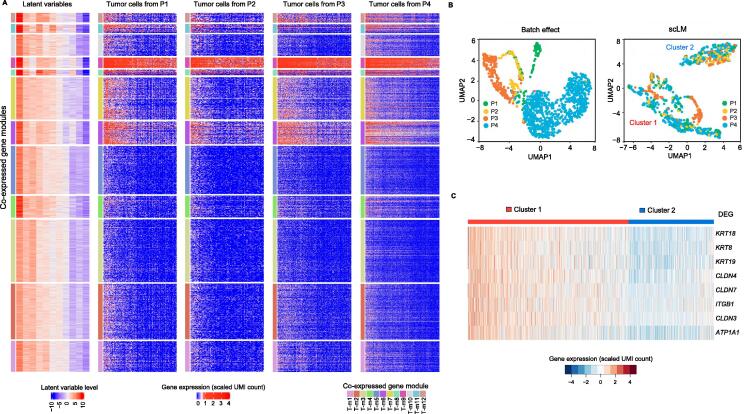
**scLM identifies co-expressed gene modules that characterize subtle cell subpopulations** **A.** Simultaneous and consensus clustering of genes across lung tumor cells from four patients (P1–P4). scLM reveals 12 co-expressed gene modules characterized by the latent variables as well as shown in gene expression data of four patients. In each heatmap, rows are genes assigned to 12 modules. In each co-expressed gene module, genes are consistently over-expressed (red) or under-expressed (blue). **B.** The left panel shows strong batch effects of different patients. The right panel depicts the UMAP visualization of single cells characterized by the co-expressed gene modules. Two evenly distributed clusters (cluster 1 and cluster 2) are identified. Different patients are distinguished by colors. **C.** Heatmap shows the differential expression pattern of EMT-related genes between two clusters. UMI, unique molecular identifier; UMAP, uniform manifold approximation and projection; DEG, differentially expressed gene.

In real-world scenarios, samples from different patients or different data sources often demonstrated highly different cell numbers, largely due to strong batch effects and technical issues. The scLM method was designed to address such highly unbalanced data that outperformed other competitors on such datasets. To validate the effectiveness of scLM, we intentionally selected patient samples that varied with respect to cell number, which could create challenges for this method. As a case study, we used scLM to analyze our in-house scRNA-seq profiling from 4 NSCLC patients (P1–P4) [41] to identify the co-expressed genes in tumor and normal epithelial cells, respectively. In tumor cells (Figure 5A, heatmap of latent variables), we discovered 12 co-expressed gene modules in the latent space (T-m1–T-m12). These modules showed clear differences but were consistently concordant across patients (Figure 5A, heatmaps of P1–P4), even though the single cells from different patients presented strong heterogeneity and batch effects (Figure 5B, left panel).

Using the 12 co-expression modules, the single cells were separated into two major clusters. In each cluster, cells from different patients mixed well without interference from batch effects (Figure 5B, right panel), which further supported that the co-expression modules were consistent across patients and not affected by batch effects. Interestingly, we found that cluster 1 had higher expression of epithelial functional markers (EMT-related genes) than cluster 2 (Figure 5C). These results indicated that co-expression modules were capable of characterizing specific cell phenotypes.

Similarly, in normal single cells, we observed 13 co-expressed gene modules (N-m1–N-m13) that showed concordant expression across individual patients (Figure S2). Then we compared the co-expressed gene modules identified from tumor and normal cells. Four modules (T-m1, T-m3, T-m4, and T-m10) were not correlated with any normal modules, suggesting that they were tumor-specific (File S1; Figure S3).

### scLM identified a common program across three types of cancer

To explore the underlying mechanism of carcinogenesis, we next extended the application of scLM to HNSCC and melanoma. In addition to the 12 co-expressed gene modules identified in NSCLC, we identified 11 modules in HNSCC and 14 modules in melanoma. To determine the similarities of these co-expression modules, we performed a pair-wise comparison using weighted Jaccard similarity, followed by hierarchical clustering. As shown in the diagram (Figure S4A), we found that most branches were dominated by a mixture of cancer types. Importantly, we identified a branch with high similarity among T-m9, HNSCC-m7, and Melanoma-m12 modules.

These three similar modules substantially overlapped with 91 genes, which were defined as a common program across three cancer types. To gain insights into the biological functions of the common program, we performed enrichment analysis in the Hallmark database (Figure S4B). The *MYC* targets v1 and hypoxia were the top enriched terms, involving the genes *FOS*, *GAPDH*, *HLA-A*, and *NFKB1A*, which suggested the common oncogenesis mechanism regardless of cancer types (File S1).

From the applications of scLM, we see three meaningful use of scLM to scientific research. 1) scLM identifies co-expressed genes that reveal novel biological processes. An example is the lung tumor-specific module that highlights cell–cell communication in tumor microenvironment (File S1; Figure S3). 2) scLM contributes to the subtle characterization of cell states. In lung cancer, scLM identifies 12 co-expression modules that are consistent across patients. These co-expression modules separate cells into two major clusters, of which one cluster presents different EMT activity suggesting more precise characterization of cell states (Figure 5). 3) With the co-expressed genes identified by scLM, both specific and common gene modules can further be explored for their translational and biological relevance. For example, in melanoma, scLM identifies two co-expressed gene modules that are associated with immune checkpoint inhibitor (ICI) resistance, which provides potential value for predicting ICI therapy response. We also find a common co-expression module from three different cancer types, and reveal the *MYC* targets and hypoxia as the common intrinsic mechanisms of tumor malignancy (File S1; Figure S4).

Given the merits of scLM, several potential limitations warrant further study. First, zero-inflated genes are excluded during pre-processing. The main reason is that, genes with inflated zeros are not informative and have negligible meaningful contribution to co-expression. The other reason is, with the fast advance of scRNA-seq technology, zero-inflation issue will be very minimal in near future. Second, in future work, we will examine the necessity of providing zero-inflation models, which specifically deal with data of poor sequencing depth and strong dropout effects. Third, the computational cost of scLM can be further reduced. We have already utilized C programming and parallel computing to dramatically boost the efficiency of scLM. However, considering that scRNA-seq data are growing into million-cell level, we will explore the use of GPU computing and cloud-based approaches to catch up with the scale of future scRNA-seq data.

## Conclusion

Co-expressed genes with coordinate expression indicate functional linkages between genes. Genes with coordinate biological functions are frequently co-transcribed, resulting in co-expression profiles. Thus, co-expressed genes can be used to intuitively associate genes with biological processes, to reveal disease-related genes, and to discern transcriptional regulatory mechanisms. Accumulative evidence supports the reliability of co-expression analysis for annotating and inferring gene functions [1], [2], [3], [9], [10], [11], [12]. Recent advances in scRNA-seq technologies enable the systematic interrogation of gene co-expression modules in specific cell types, and the elucidation of the underlying biological mechanisms [13], [54], [55]. The improved data resolution and quality allow accurate identification of disease-related modules and regulatory genes for specific cell types and specific tissues. Thus, we expect co-expression analysis to be more widely applied due to the technology advances.

In this study, we introduce a novel method, scLM, to simultaneously identify co-expressed genes across multiple single-cell datasets. The scLM algorithm uses the conditional NB distribution with latent variables to disentangle co-expression patterns across multiple datasets. To our knowledge, scLM is the first available tool that is capable of leveraging multiple scRNA-seq datasets to accurately detect co-expressed genes. We provide an overview of scLM and illustrate how scLM can be used to further characterize cell states and identify tumor-specific modules in lung cancer. We demonstrate that the tumor-specific modules are enriched in pathways, including cell–cell communication and *SMAD2/3/4* transcriptional activity, with identified upstream transcriptional factors including *TEAD1* and *FOXA1*. We further show the clinical prognostic significance of these discoveries in clinical samples. Moreover, we explore the common co-expressed genes, *i.e.*, the common module, across three cancer types and offer intrinsic mechanisms of tumor malignancy. The common module is highly enriched in the *MYC* targets v1 and hypoxia, suggesting the presence of common intrinsic oncogenesis mechanisms. Additionally, the common module is shown to be related with clinical response to ICI in melanoma patients, suggesting that the common module provides predictive value of ICI therapy response.

Compared with other methods, scLM has several key advantages: 1) scLM accounts for data heterogeneity and variances among multiple datasets, such as unbalanced sequencing depths and technical biases in library preparation. 2) scLM leverages information across datasets for detecting stable and conserved co-expression modules with high accuracy and reproducibility. 3) scLM is an integrated pipeline that uses raw count matrix without prior batch-correction as input, thus can be easily applied to scRNA-seq data. Overall, scLM opens possibilities for further investigation and mechanistic interpretation of co-expressed genes. With the growing scRNA-seq data, scLM is poised to become a valuable tool for elucidating co-expression studies in single-cell transcriptomics.

## Ethical statement

For our in-house data, fresh remnant tumor and adjacent normal tissues were collected at the time of elective curative resection by the Tumor Tissue and Pathology Shared Resource (TTPSR) of the Wake Forest Baptist Medical Center Comprehensive Cancer Center (WFBMC-CCC), USA. Collections by the TTPSR adhere to Institutional Review Board approved procedures (Advanced Tumor Bank protocol CCCWFU 01403, TTPSR collections IRB BG04-104 which also allows for the use of de-identified protected health information along with the tissue samples). Acquisition of de-identified samples from the TTPSR for single-cell isolation and research use was in accordance with approved IRB protocol 00048977. All patients provided written consent to participate in the study, which was approved by the institutional reviewing board.

## Code availability

A user-friendly R package with all the key features of the scLM method is available at https://github.com/QSong-github/scLM.

## CRediT author statement

**Qianqian Song:** Data curation, Methodology, Software, Writing - original draft. **Jing Su:** Methodology, Software, Validation, Writing - review & editing. **Lance D. Miller:** Investigation, Writing - review & editing. **Wei Zhang:** Conceptualization, Supervision. All authors read and approved the final manuscript.

## Competing interests

The authors have declared no competing interests.

## Supplementary material

Supplementary File S1 **Applications of scLM in single cell RNA-seq data**.

Supplementary Figure S1 **Significantly enriched GO terms for different methods**. The average number of GO terms that are detected as significantly enriched at the threshold of adjusted P value < 0.01, based on the co-expressed genes identified by different methods.

Supplementary Figure S2 **scLM identifies co-expressed gene modules in lung normal cells**. scLM yields 13 co-expressed gene modules characterized by the latent variables as well as gene expression data. In each heatmap, rows are genes assigned to 13 modules. In each co-expressed gene module, genes are consistently over-expressed (red) or under-expressed (blue).

Supplementary Figure S3 **scLM uncovers tumor-specific modules enriched in specific cell subpopulations**. **A.** Heatmap depicts the pairwise correlations between the 12 co-expressed gene modules from tumor (rows) and the 13 co-expression modules from normal (columns). Red color with star represents significant association (Pearson correlation > 0.5 and *P* value < 0.05). **B.** UMAP visualization of single cells that are labeled with high versus low expression levels of the tumor-specific module T-m10. **C.** Significantly enriched pathways in the Reactome database are identified based on the tumor-specific modules (T-m1, T-m3, T-m10). The *x*-axis represents the enrichment score defined by –log10(adjusted *P* value) of the enrichment test. **D**. Putative upstream regulators of T-m10 are identified and labeled as blue. E. KM survival curves for TCGA patients with lung squamous cell cancer (*n* = 545), stratified by average expression (top 30% versus bottom 30%) of the tumor-specific module T-m10. Log-rank test *P* value is shown. The *y*-axis represents the probability of overall survival, and the *x*-axis represents time in Months.

Supplementary Figure S4 **scLM identifies a common program across three cancer types**. **A.** The dendrogram presents the similarity of the co-expression modules from three cancer types, including 12 co-expressed gene modules from NSCLC, 11 from HNSCC, and 14 from melanoma. **B.** Bar plots show the significantly enriched processes of the common program in Hallmark database. The *x*-axis represents the enrichment score defined by –log10(adjusted P value) of the enrichment test.

Supplementary Figure S5 **Tumor-specific modules are enriched in specific cell subpopulations**. UMAP visualization of single cells labeled by different expression level (high versus low) of the tumor-specific modules (T-m1, T-m3, T-m4).

Supplementary Figure S6 **Biological mechanism underlying the tumor-specific modules**. **A.** and **B.** Significantly enriched processes in the KEGG (A) and Hallmark (B) databases are identified based on the tumor-specific modules (T-m1, T-m3, T-m4, and T-m10). The *x*-axis represents the enrichment score defined by –log10(adjusted P value) of the enrichment test. C. Putative upstream regulators of the tumor-specific modules (T-m1, T-m3, and T-m4) are identified and labeled as blue.

Supplementary Figure S7 **Prognostic significance of the tumor-specific modules**. **A.** KM survival curves of TCGA LUSC patients, which are stratified by the average expression (top 30% versus bottom 30%) of tumor-specific modules (T-m1, T-m3, and T-m4). **B.** KM survival curves of TCGA LUAD patients that are stratified by the average expression (top 30% versus bottom 30%) of tumor-specific modules (T-m1, T-m3, T-m4, and T-m10). Log-rank test *P* values are shown. The *y*-axis represents the probability of overall survival, and the *x*-axis represents time in Months.

Supplementary Figure S8 **A common program across three cancer types**. **A.** The left panel shows strong batch effects among different patients. The right panel depicts the UMAP visualization of single cells characterized by co-expressed gene modules. Different patients are distinguished by color. The upper part shows the melanoma data, whereas the lower part shows the HNSCC data. **B.** Boxplots shows the expression levels of three similar modules (i.e., HNSCC-m7, T-m9, Melanoma-m12) in their corresponding tumor samples versus normal samples from TCGA. C. KM curves show that HNSCC-m7 and Melanoma-m12 are associated with poor overall survival in HNSCC and melanoma patients, respectively.

Supplementary Figure S9 **Co-expressed gene modules are potential markers of immunotherapy response**. Melanoma-m5 and Melanoma-m9 distinguish the PR patients from the CR patients in an external patient cohort that are received with immunotherapy treatment.
