# Supplementary material for: scLM: Automatic Detection of Consensus Gene Clusters Across Multiple Single-cell Datasets
Source: Genomics Proteomics Bioinformatics. 2020 Dec 24;19(2):330–41. doi: 10.1016/j.gpb.2020.09.002 (PMC8602751; doi:10.1016/j.gpb.2020.09.002)
Supplement: Supplementary File S1 — Applications of scLM in single cell RNA-seq data. [file mmc1.docx]

**File S1 Applications of scLM in single cell RNA-seq data**

**Case study 1: scLM identified tumor-specific modules enriched in specific cell state**

As a case study, we used scLM to analyze our in-house scRNA-seq profiling from 4 NSCLC patients (P1–P4) to identify co-expressed genes in tumor and normal epithelial cells respectively. In tumor cells (Figure 5A, heatmap of latent variables), we discovered 12 co-expressed gene modules in the latent space (T-m1–T-m12). These modules showed clear differences but were consistently concordant across patients (Figure 5A, heatmaps of P1–P4), even though the single cells from different patients presented strong heterogeneity and batch effects (Figure 5B, left panel).

Using the 12 co-expression modules, the single cells were separated into two major clusters. In each cluster, cells from different patients mixed well without interference from batch effects (Figure 5B, right panel), which further supported that the co-expression modules were consistent across patients and not affected by batch effects. Interestingly, we found that cluster 1 had higher expression of epithelial functional markers (EMT-related genes) than cluster 2 (Figure 5C). These results indicated that co-expression modules were capable of characterizing specific cell phenotypes.

Similarly, in normal single cells, we observed 13 co-expressed gene modules (N-m1–N-m13) that showed concordant variations in expression levels across individual patients (Figure S2). Further comparison of co-expression modules from tumor and normal cells revealed very dissimilar patterns (Figure S3A). Some tumor modules (*e.g.*, T-m11 and T-m5) had correlations with one normal module, whereas other tumor modules (*e.g.*, T-m2, T-m6, and T-m7) were associated with more than two normal modules. Four tumor modules (T-m1, T-m3, T-m4, and T-m10) were not correlated with any normal modules, suggesting they were tumor specific. Meanwhile, we found that these tumor-specific modules had higher expression levels in cluster 1 than in cluster 2 (T-m10 is shown in Figure S3B; T-m1, T-m3, and T-m4 are shown in Figure S5).

To better understand these tumor-specific modules, we examined their enriched biological categories in the REACTOME (Figure S3C), KEGG and Hallmark databases (Figure S6A–B). T-m10 was enriched in cell-cell communication, adherence junction, and leukocyte transendothelial migration pathways. Genes in T-m1 were associated with the circadian clock and the circadian rhythm mammal pathways. T-m3 was enriched with downregulation of *SMAD2/3/4* transcriptional activity and ubiquitin-mediated proteolysis pathways. T-m4 was found with enrichment in Hallmark database only. Next, we identified the putative upstream transcriptional factor (TF) that mediated these tumor-specific modules (Figure S3D, S6C). Each module was subjected to cis-regulatory motif analysis using the RcisTarget tool to identify their upstream TFs, and only those involved in co-expression modules were retained. *TEAD1* and *FOXA1* were the major co-expressed regulators of the T-m10 module. *YY1* and *ELF2* were regulators of the T-m1 and T-m3 modules, respectively, while *SRF* and *POLR2A* regulated the T-m4 module. To further investigate whether these tumor-specific modules have prognostic significance in clinical samples, we used the NSCLC patient data from The Cancer Genome Atlas (TCGA). Remarkably, in patients with Lung Squamous Cell Carcinoma (LUSC), we observed significant associations between overall survival and each tumor-specific module (T-m10 was shown in Figure S3E; T-m1, T-m3, and T-m4 were shown in Figure S7A). However, such associations were not seen in patients with lung adenocarcinoma (LUAD) (Figure S7B).

**Case study 2: scLM identified a common program across three types of cancer**

Tumors located in different organs carried different genomic and molecular characteristics but also shared common hallmarks that were intrinsic for carcinogenesis. To explore such common hallmarks across different tumor types, we next extended our analysis to the head and neck squamous cell carcinoma (HNSCC) and melanoma, by applying the scLM method to the corresponding public scRNA-seq data. In addition to the 12 co-expression modules in NSCLC, we identified 11 modules in HNSCC and 14 modules in melanoma.

Not only in lung cancer, the single cell data from melanoma and HNSCC also showed strong batch effects (Figure S8A, left panel). The co-expressed genes identified by scLM separated these single cells into major clusters. In each cluster, cells from different patients mixed well without interference from batch effects (Figure S8A, right panel). For both melanoma and HNSCC, the co-expressed genes identified by scLM still achieved to align cells evenly that were not interfered with batch effects from different patients. These results further confirmed the consensus properties of the co-expressed genes identified by scLM.

To determine the (dis)similarities among the co-expression modules from these three cancer types, we performed a pair-wise comparison of these modules using weighted jaccard similarity, followed by hierarchical clustering. As shown in the diagram (Figure S4A), we found that most branches were dominated by a mixture of cancer types instead of one specific cancer type. For example, one branch contained module 1 from melanoma (Melanoma-m1), module 3 from NSCLC (T-m3), and module 3 from HNSCC (HNSCC-m3); whereas another branch contained melanoma-m14, T-m5, and HNSCC-m3. No branch was dominated by a single cancer type, indicating that most modules are not cancer specific. Importantly, we identified a distinct branch with high similarity among T-m9, HNSCC-m7 and Melanoma-m12 modules.

Since the three modules from different cancer types had high similarity, we investigated their potential clinical value in TCGA data (Figure S8B-C). In both LUAD and LUSC subtypes of lung cancer, the T-m9 showed significantly higher expression in tumor than normal samples. In HNSCC samples, the HNSCC-m7 was also higher in tumor samples than normal samples. Melanoma module 12 (Melanoma-m12) was more pronounced in uveal melanoma (UVM) samples than in skin cutaneous melanoma (SKCM) samples from TCGA. In both HNSCC and uveal melanoma cases, these modules (HNSCC-m7 and Melanoma-m12) were associated with poorer survival in patients (with significant *P* values of 2.5e-03 and 5.0e-03, respectively). We did not find significant survival difference associated with the corresponding T-m9 in LUAD and LUSC cases. Overall, these results demonstrated the prevalent malignancy properties of these three similar modules across three cancer types.

These three similar modules substantially overlapped with 91 genes, which were defined as a common program across three cancer types. To gain insights into the biological functions of the common program, we performed enrichment analysis in the Hallmark database (Figure S8D). The *MYC* targets v1 and hypoxia were the top enriched terms, involving the genes *FOS*, *GAPDH*, *HLA-A*, and *NFKB1A*. This result suggests the presence of a common intrinsic mechanism of tumor malignancy regardless of cancer types.

With the co-expression modules identified by scLM, it prompted us to test whether the co-expression modules were related with clinical responses to immune checkpoint inhibitors (ICI). Therefore, we looked into the available RNA-seq cohort collected from 112 melanoma patients prior to ICI treatment (Figure S9), to determine the ICI resistance associated co-expression modules in melanoma. Through comparing the post-ICI-complete response (CR) patients to post-ICI-partial response (PR) patients, we found that the Melanoma-m5 and Melanoma-m9 significantly distinguished the ICI-CR from the ICI-PR patients. It suggested that these two modules related with ICI resistance that might provide predictive value of ICI therapy response.
