# Supplementary figures and images for: scLM: Automatic Detection of Consensus Gene Clusters Across Multiple Single-cell Datasets
Source: Genomics Proteomics Bioinformatics. 2020 Dec 24;19(2):330–41. doi: 10.1016/j.gpb.2020.09.002 (PMC8602751; doi:10.1016/j.gpb.2020.09.002)

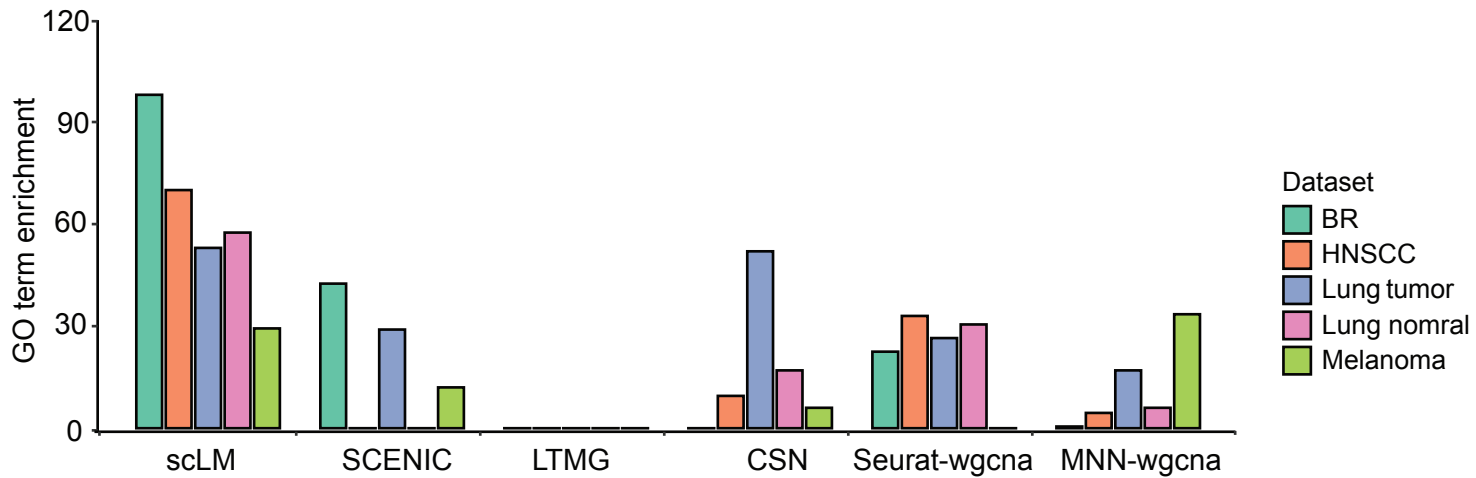

Supplement: Supplementary Figure S1 — Significantly enriched GO terms for different methods. The average number of GO terms that are detected as significantly enriched at the threshold of adjusted P value < 0.01, based on the co-expressed genes identified by different methods. [file mmc2.pdf]

A

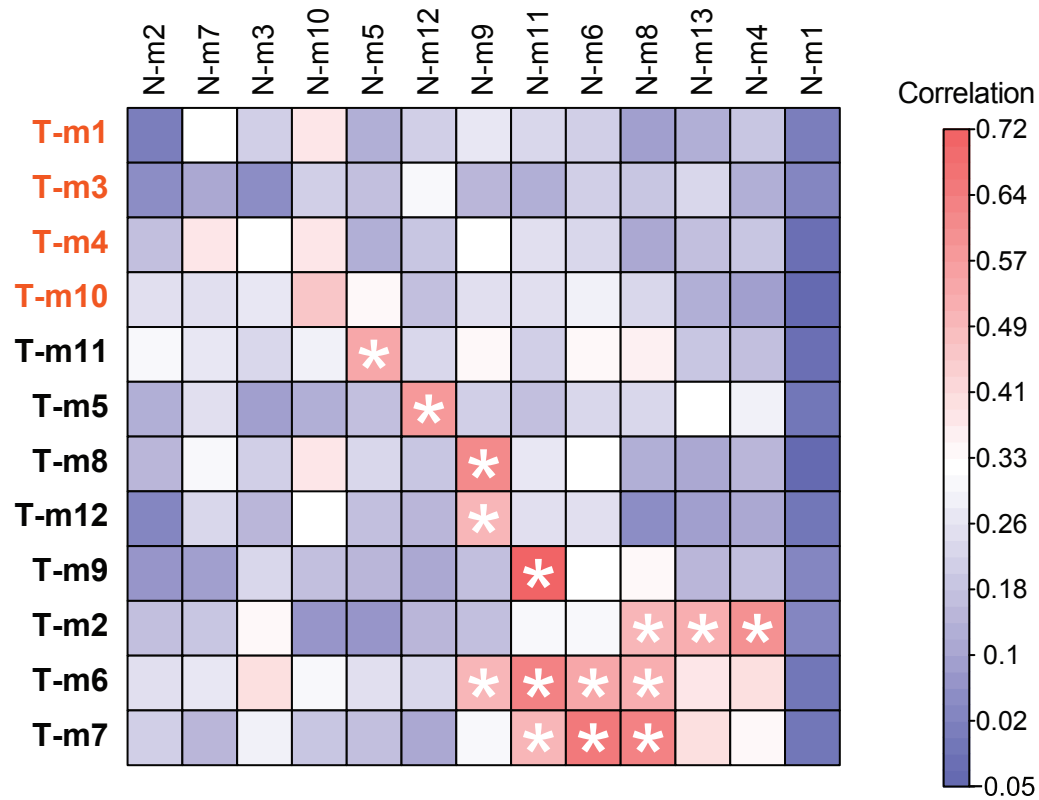

B

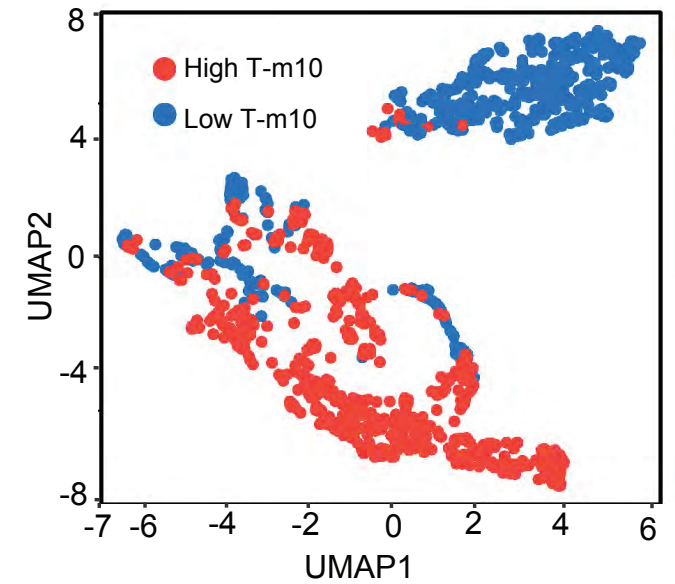

D

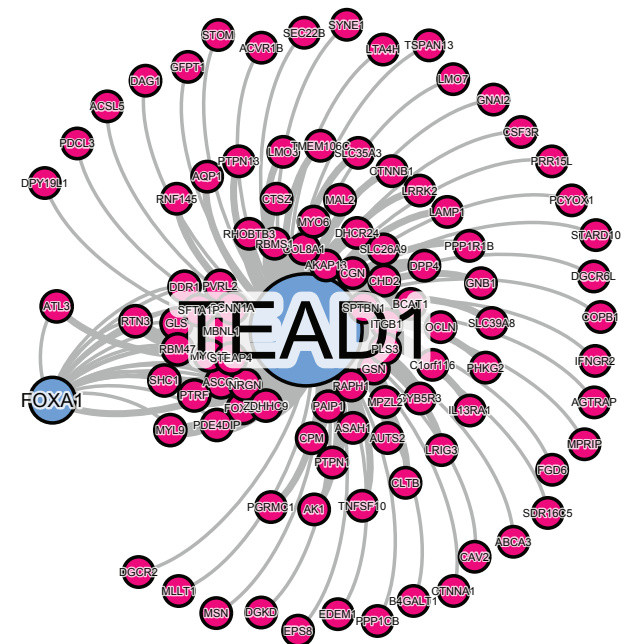

C

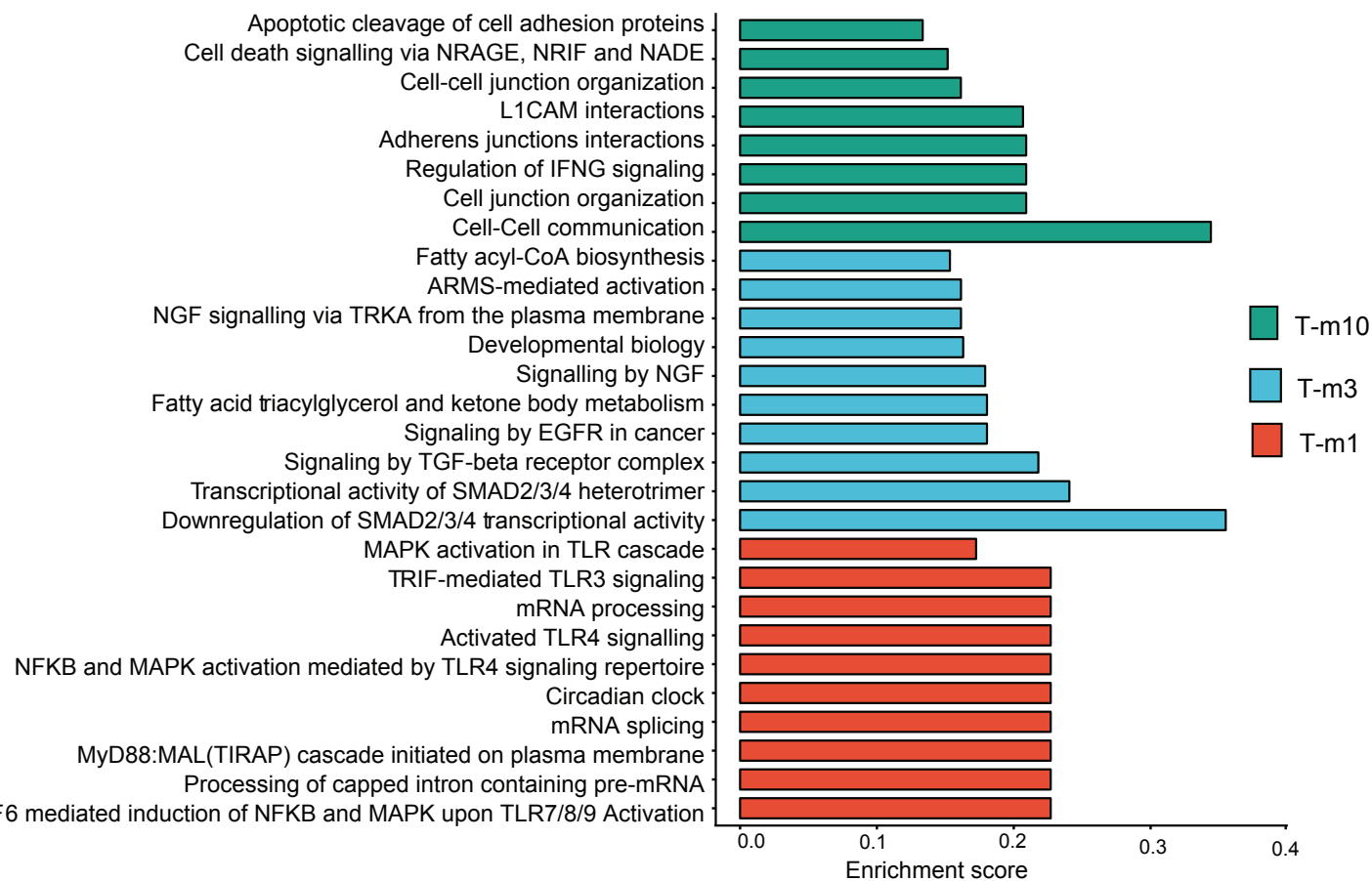

E

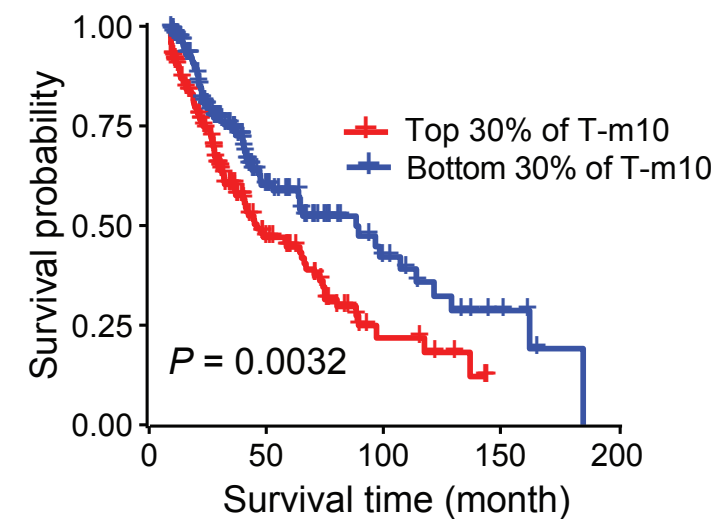

Supplement: Supplementary Figure S3 — scLM uncovers tumor-specific modules enriched in specific cell subpopulations. A. Heatmap depicts the pairwise correlations between the 12 co-expressed gene modules from tumor (rows) and the 13 co-expression modules from normal (columns). Red color with star represents significant association (Pearson correlation > 0.5 and P value < 0.05). B. UMAP visualization of single cells that are labeled with high versus low expression levels of the tumor-specific module T-m10. C. Significantly enriched pathways in the Reactome database are identified based on the tumor-specific modules (T-m1, T-m3, T-m10). The x-axis represents the enrichment score defined by –log10(adjusted P value) of the enrichment test. D. Putative upstream regulators of T-m10 are identified and labeled as blue. E. KM survival curves for TCGA patients with lung squamous cell cancer (n = 545), stratified by average expression (top 30% versus bottom 30%) of the tumor-specific module T-m10. Log-rank test P value is shown. The y-axis represents the probability of overall survival, and the x-axis represents time in Months. [file mmc4.pdf]

**A**

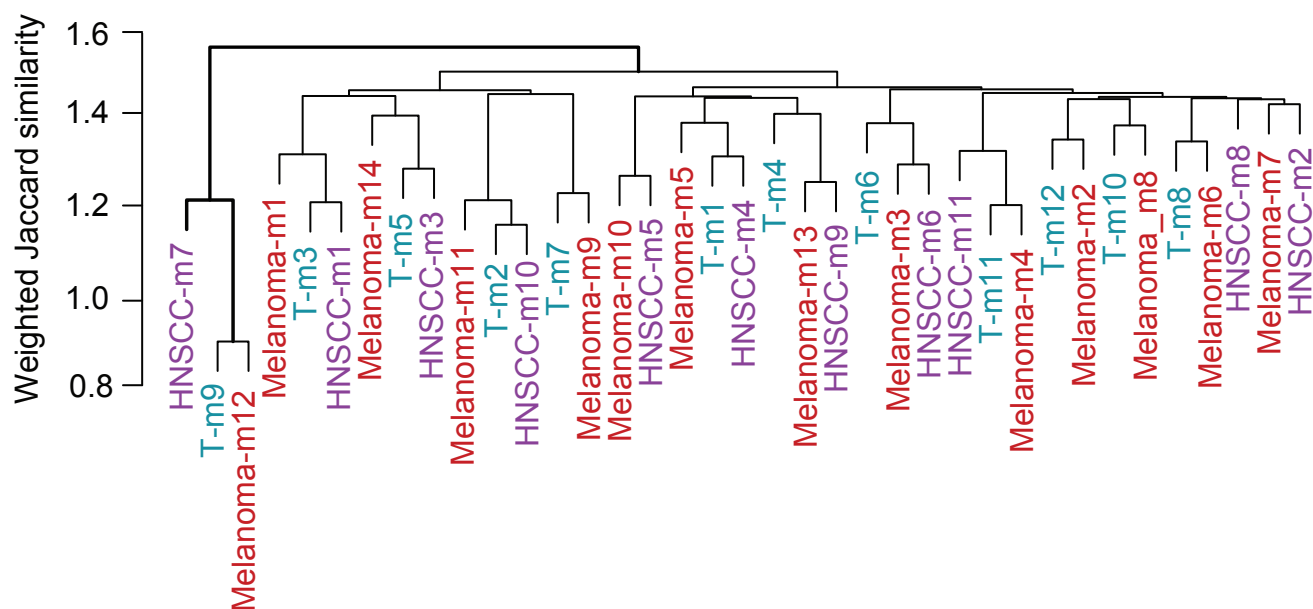

**B**

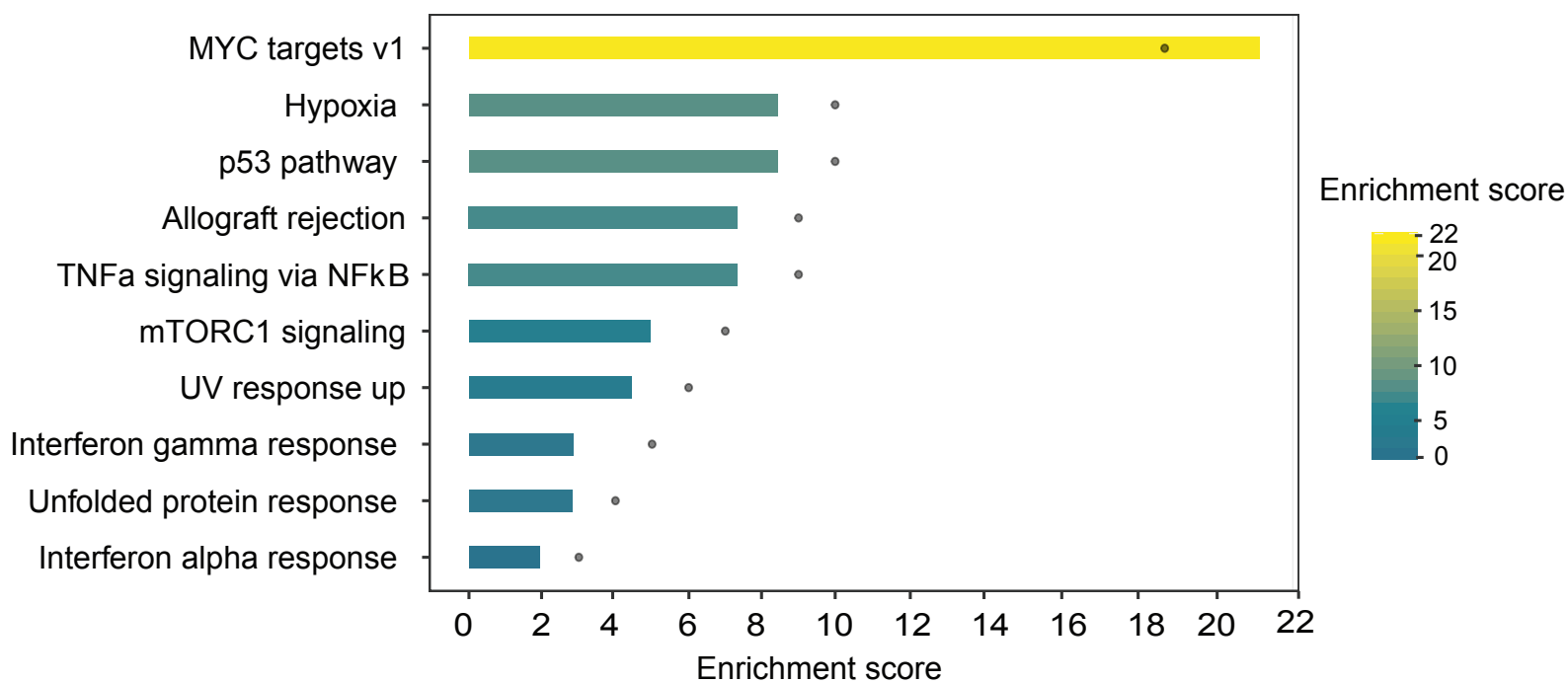

Supplement: Supplementary Figure S4 — scLM identifies a common program across three cancer types. A. The dendrogram presents the similarity of the co-expression modules from three cancer types, including 12 co-expressed gene modules from NSCLC, 11 from HNSCC, and 14 from melanoma. B. Bar plots show the significantly enriched processes of the common program in Hallmark database. The x-axis represents the enrichment score defined by –log10(adjusted P value) of the enrichment test. [file mmc5.pdf]

T-m1

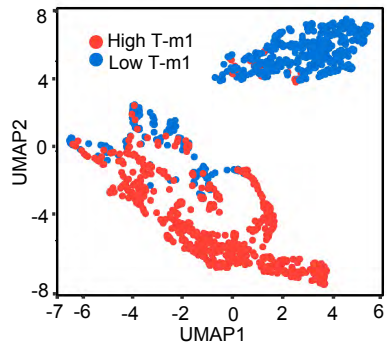

T-m3

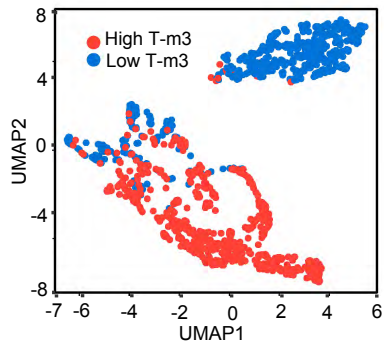

T-m4

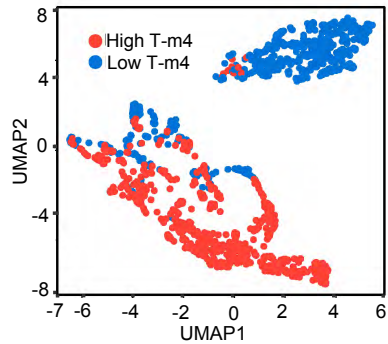

Supplement: Supplementary Figure S5 — Tumor-specific modules are enriched in specific cell subpopulations. UMAP visualization of single cells labeled by different expression level (high versus low) of the tumor-specific modules (T-m1, T-m3, T-m4). [file mmc6.pdf]

A KEGG pathway enrichment

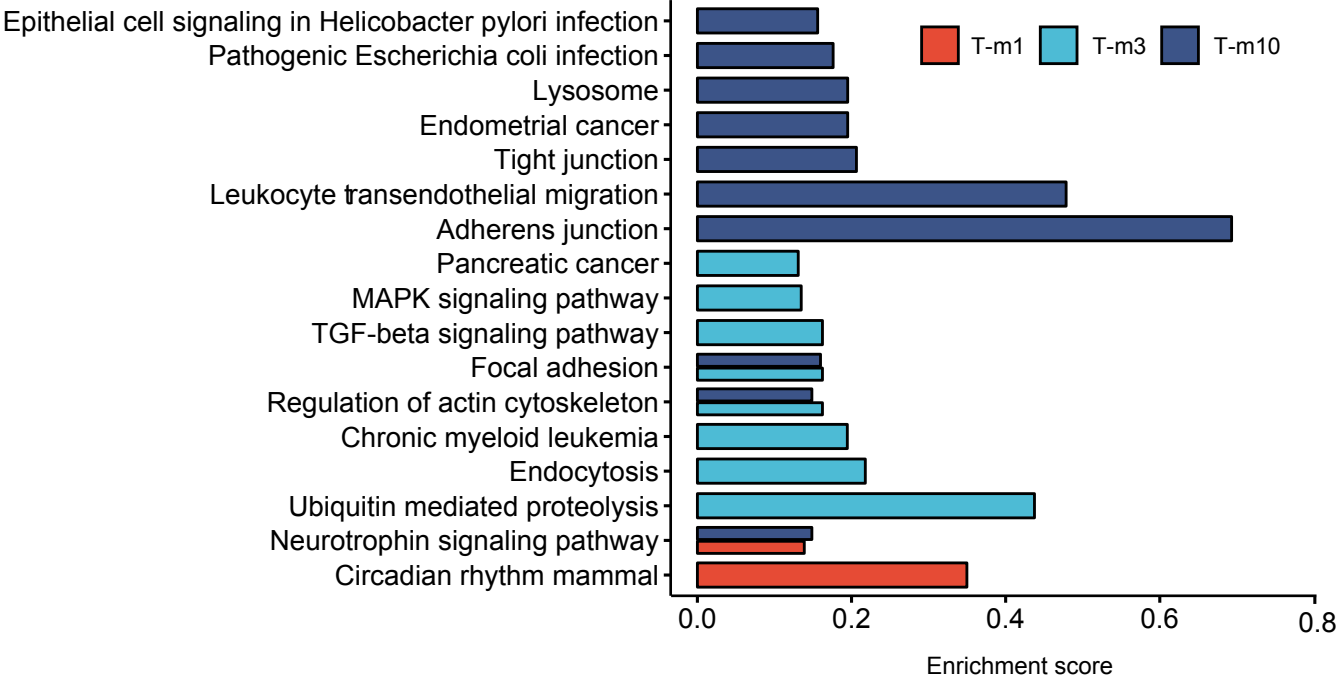

B Reactome pathway enrichment

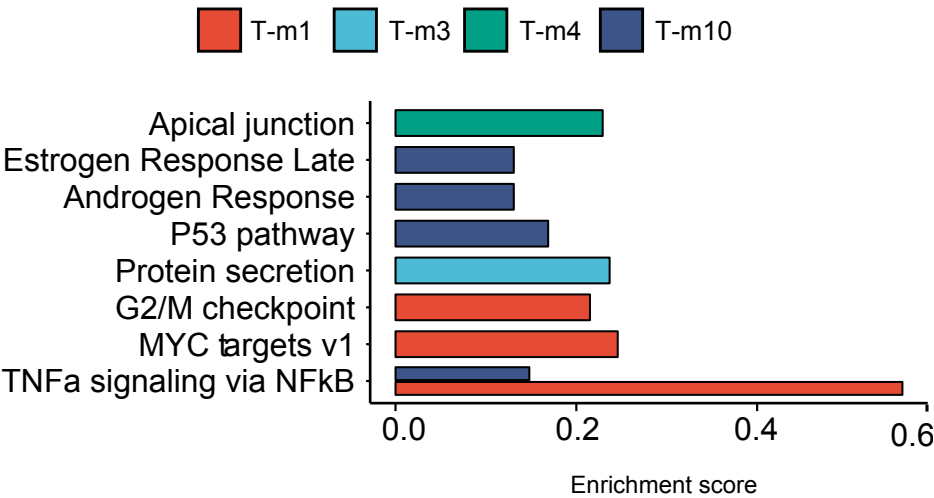

C

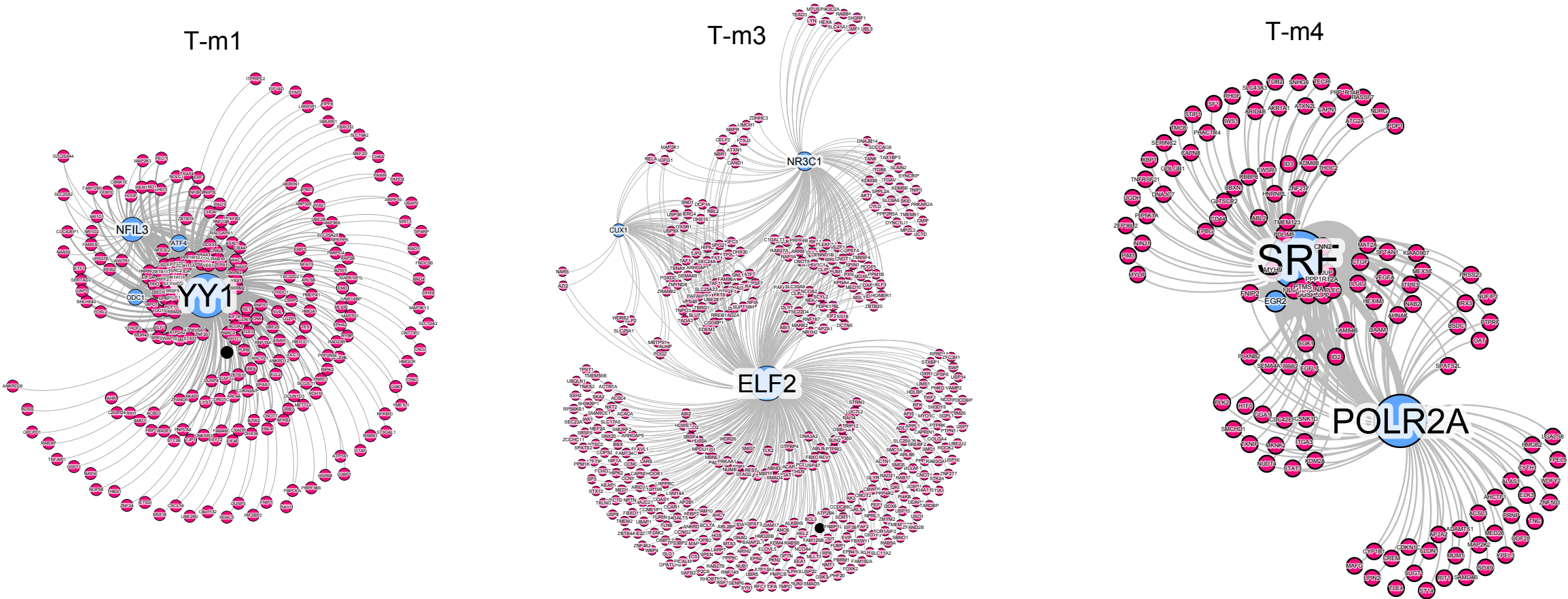

Supplement: Supplementary Figure S6 — Biological mechanism underlying the tumor-specific modules. A. and B. Significantly enriched processes in the KEGG (A) and Hallmark (B) databases are identified based on the tumor-specific modules (T-m1, T-m3, T-m4, and T-m10). The x-axis represents the enrichment score defined by –log10(adjusted P value) of the enrichment test. C. Putative upstream regulators of the tumor-specific modules (T-m1, T-m3, and T-m4) are identified and labeled as blue. [file mmc7.pdf]

**A**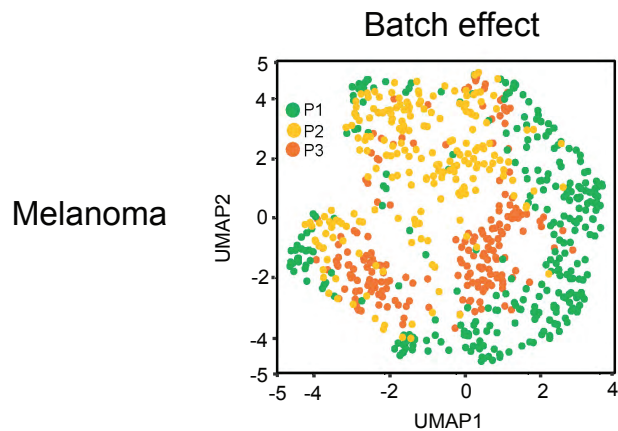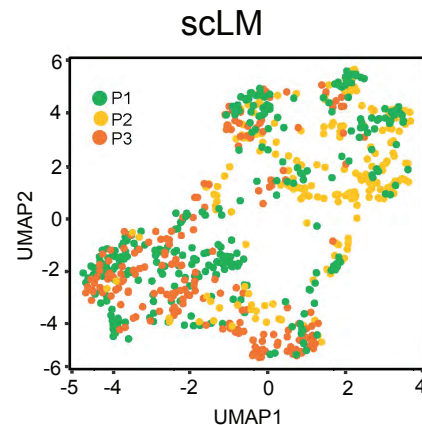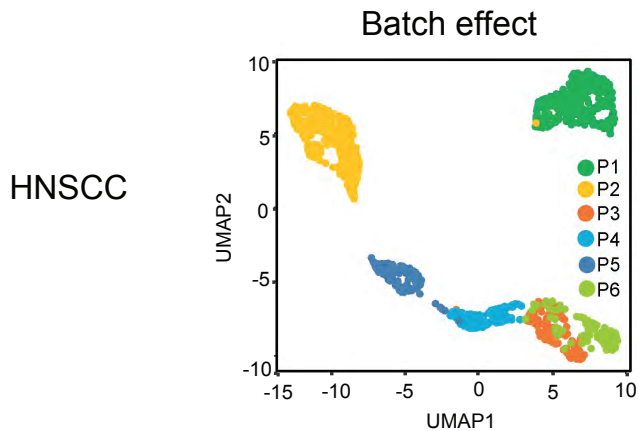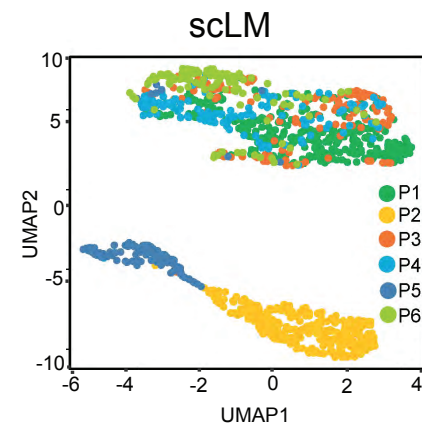**B**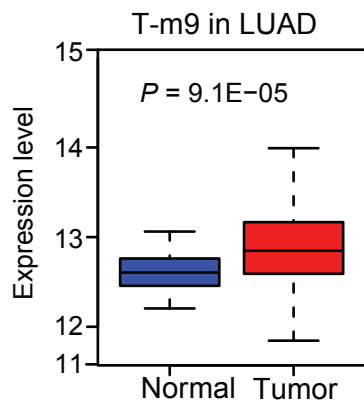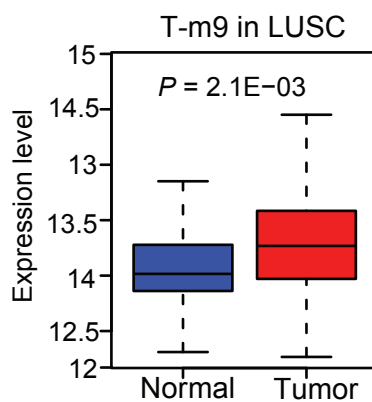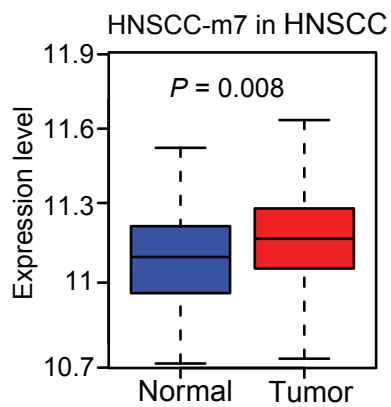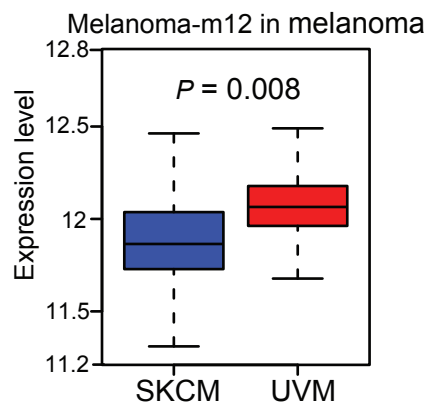**C**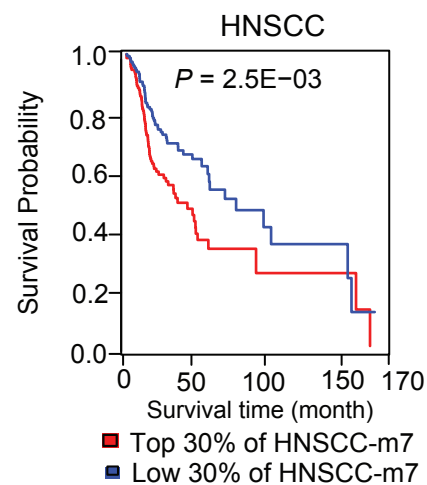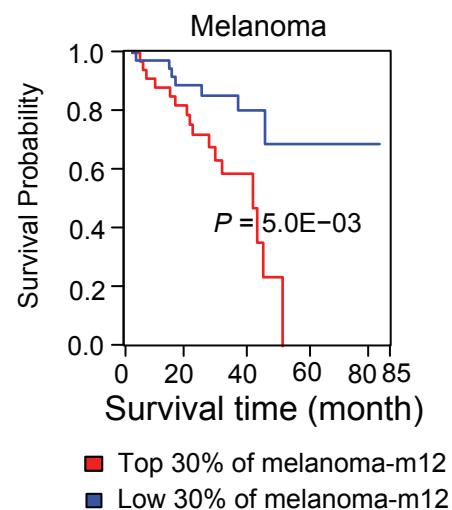

Supplement: Supplementary Figure S8 — A common program across three cancer types. A. The left panel shows strong batch effects among different patients. The right panel depicts the UMAP visualization of single cells characterized by co-expressed gene modules. Different patients are distinguished by color. The upper part shows the melanoma data, whereas the lower part shows the HNSCC data. B. Boxplots shows the expression levels of three similar modules (i.e., HNSCC-m7, T-m9, Melanoma-m12) in their corresponding tumor samples versus normal samples from TCGA. C. KM curves show that HNSCC-m7 and Melanoma-m12 are associated with poor overall survival in HNSCC and melanoma patients, respectively. [file mmc9.pdf]

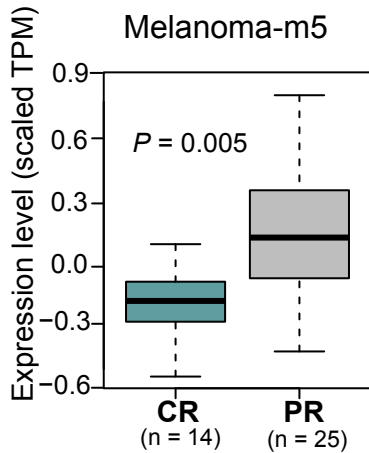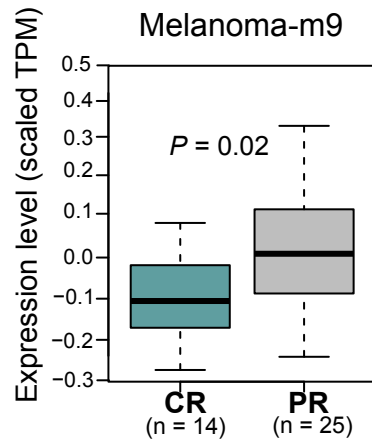

Supplement: Supplementary Figure S9 — Co-expressed gene modules are potential markers of immunotherapy response. Melanoma-m5 and Melanoma-m9 distinguish the PR patients from the CR patients in an external patient cohort that are received with immunotherapy treatment. [file mmc10.pdf]
