# Supplementary material for: scLM: Automatic Detection of Consensus Gene Clusters Across Multiple Single-cell Datasets
Source: Genomics Proteomics Bioinformatics. 2020 Dec 24;19(2):330–41. doi: 10.1016/j.gpb.2020.09.002 (PMC8602751; doi:10.1016/j.gpb.2020.09.002)
Supplement: Supplementary Figure S2 — scLM identifies co-expressed gene modules in lung normal cells. scLM yields 13 co-expressed gene modules characterized by the latent variables as well as gene expression data. In each heatmap, rows are genes assigned to 13 modules. In each co-expressed gene module, genes are consistently over-expressed (red) or under-expressed (blue). [file mmc3.pdf]

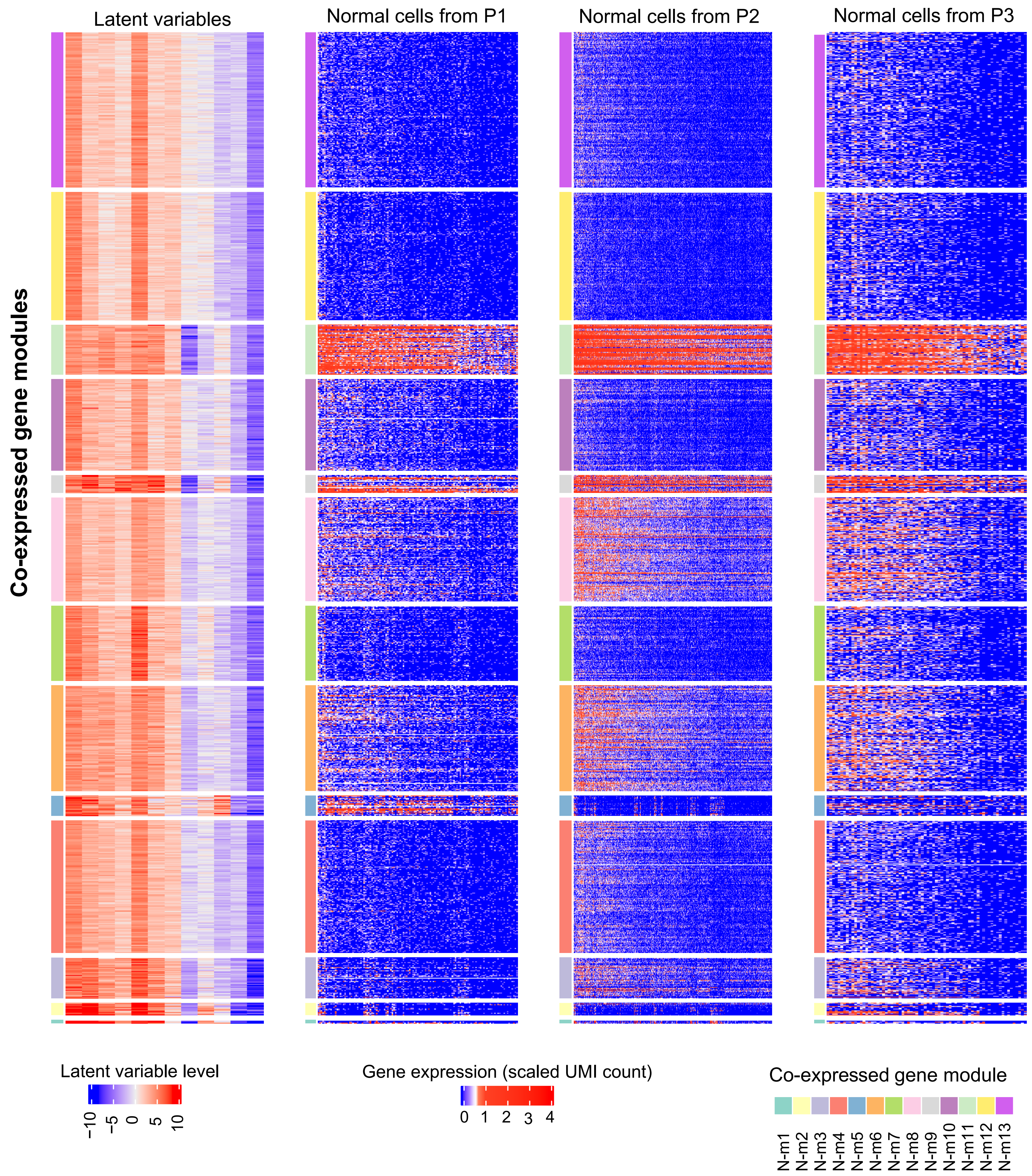

Latent variables
Normal cells from P1
Normal cells from P2
Normal cells from P3
Co-expressed gene modules
Latent variable level
−10 −5 0 5 10
Gene expression (scaled UMI count)
0 1 2 3 4
Co-expressed gene module
N-m1
N-m2
N-m3
N-m4
N-m5
N-m6
N-m7
N-m8
N-m9
N-m10
N-m11
N-m12
N-m13
