# Supplementary material for: scLM: Automatic Detection of Consensus Gene Clusters Across Multiple Single-cell Datasets
Source: Genomics Proteomics Bioinformatics. 2020 Dec 24;19(2):330–41. doi: 10.1016/j.gpb.2020.09.002 (PMC8602751; doi:10.1016/j.gpb.2020.09.002)
Supplement: Supplementary Figure S7 — Prognostic significance of the tumor-specific modules. A. KM survival curves of TCGA LUSC patients, which are stratified by the average expression (top 30% versus bottom 30%) of tumor-specific modules (T-m1, T-m3, and T-m4). B. KM survival curves of TCGA LUAD patients that are stratified by the average expression (top 30% versus bottom 30%) of tumor-specific modules (T-m1, T-m3, T-m4, and T-m10). Log-rank test P values are shown. The y-axis represents the probability of overall survival, and the x-axis represents time in Months. [file mmc8.pdf]

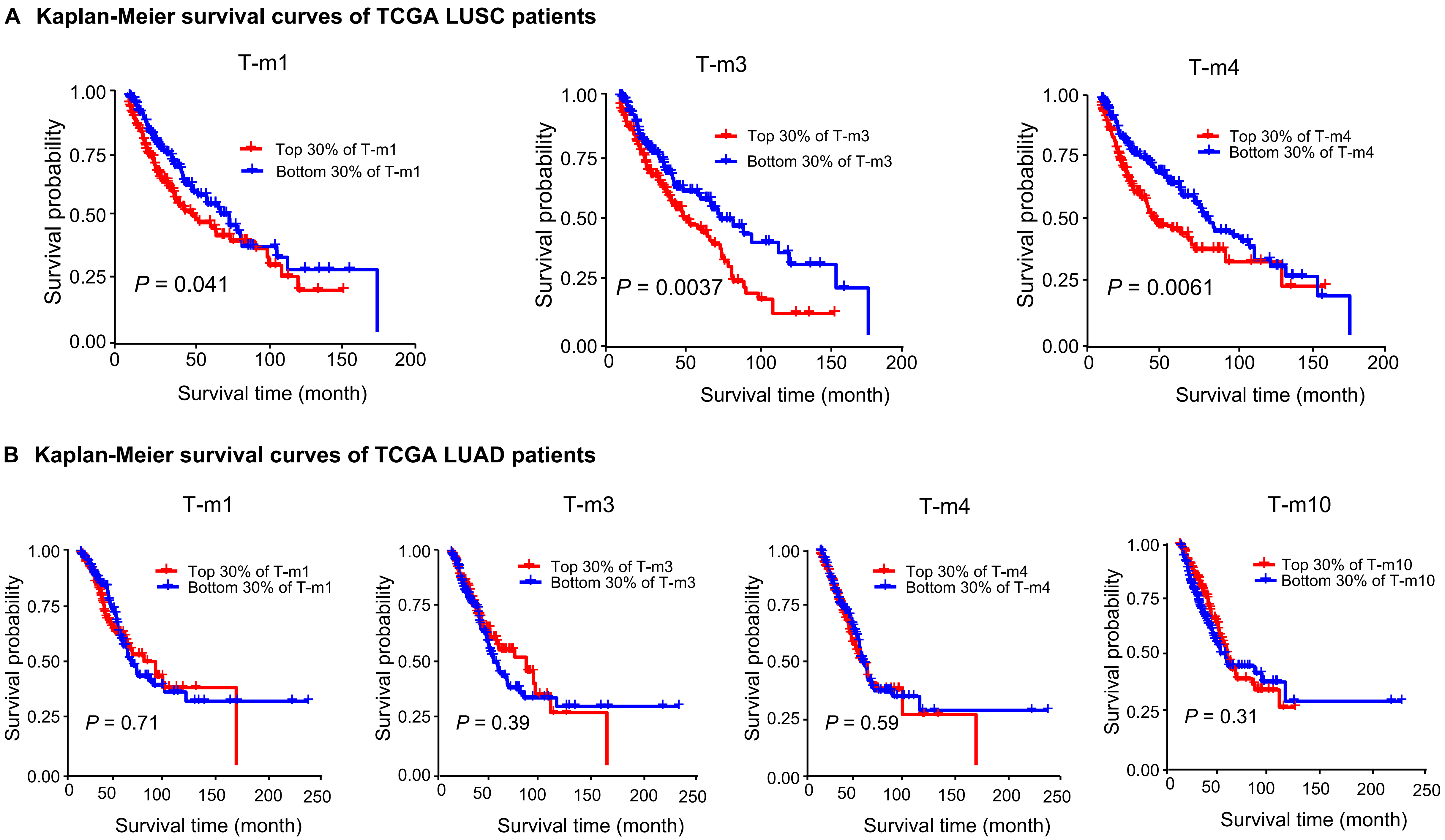
A Kaplan-Meier survival curves of TCGA LUSC patients
T-m1
Top 30% of T-m1
Bottom 30% of T-m1
P = 0.041
T-m3
Top 30% of T-m3
Bottom 30% of T-m3
P = 0.0037
T-m4
Top 30% of T-m4
Bottom 30% of T-m4
P = 0.0061
Survival probability
Survival time (month)
B Kaplan-Meier survival curves of TCGA LUAD patients
T-m1
Top 30% of T-m1
Bottom 30% of T-m1
P = 0.71
T-m3
Top 30% of T-m3
Bottom 30% of T-m3
P = 0.39
T-m4
Top 30% of T-m4
Bottom 30% of T-m4
P = 0.59
T-m10
Top 30% of T-m10
Bottom 30% of T-m10
P = 0.31
Survival probability
Survival time (month)
